# TRPC3‐Driven Calcium Microdomains Instruct Peripheral Nerve Regeneration With Protective Implications for Central Neurons

**DOI:** 10.1002/advs.77156

**Published:** 2026-08-25

**Authors:** Yu Lu, Jianbo Zhao, Zhenli Xie, Yuan Wang, Ruolin Wang, Bianbian Wang, Chuhan Shen, Yali Chen, Jingyu Yao, Ziyang Li, Huadong Xu, Zuying Chai, Zhuoyu Zhang, Changhe Wang, Rong Huang

**Affiliations:** ^1^ Neuroscience Research Center Key Laboratory of Biomedical Information Engineering of Ministry of Education School of Life Science and Technology Xi'an Jiaotong University Xi'an China; ^2^ Department of Neurology Xiongan Xuanwu Hospital Xiongan New Area China; ^3^ School of Medicine Shanghai Jiaotong University Shanghai China; ^4^ Neurological Department of Tongji Hospital School of Medicine Tongji University Shanghai China; ^5^ Key Laboratory of Medical Electrophysiology Ministry of Education of China Collaborative Innovation Center for Prevention and Treatment of Cardiovascular Disease Institute of Cardiovascular Research Southwest Medical University Luzhou China

**Keywords:** axonal regeneration, calmodulin, dopaminergic neuron protection, dorsal root ganglion neurons, peripheral and central nervous system repair, spontaneous Ca^2+^ microdomain signals, TRPC3 channel

## Abstract

A central challenge in neural repair is the “calcium paradox”: while Ca^2+^ is essential for neuronal growth, global elevations often trigger toxicity rather than repair. Spontaneous near‐membrane Ca^2+^ microdomains (smCa) have been identified in sensory neurons, yet their molecular origin and functional role in repair remain unclear. Here, by resolving Ca^2+^ dynamics at sub‐cellular resolution, we show that TRPC3 drives smCa hotspots, which function as a localized signaling module that avoids global toxicity to instruct repair. Using peripheral sensory neurons as a primary model, we show that TRPC3‐smCa constitutes a basic instructive unit that appears both necessary and sufficient to initiate axon regeneration, a process that involves calmodulin‐dependent pathways. Following sciatic nerve injury, TRPC3‐smCa axis is essential for structural and functional recovery. Our data further suggest that this repair module also operates in central nervous system: in midbrain dopaminergic neurons, TRPC3‐smCa is associated with a neuroprotective state, correlating with enhanced neuronal survival and motor function in a Parkinson's disease model. This pro‐repair correlation is further observed in human ESC‐derived dopaminergic neurons. Collectively, our findings identify TRPC3‐driven Ca^2+^ microdomains as a repair module, pointing to a spatial logic for neural repair with potential relevance for nerve injury and neurodegenerative disorders.

## Graphical abstract

How localized Ca^2+^ signals orchestrate neural repair remains unclear. Here, we show that TRPC3‐driven calcium microdomains direct nerve repair by coupling spatially confined Ca^2+^ signals to axon regeneration. This module operates in injured peripheral neurons and correlates with neuroprotection in Parkinson's disease, revealing a spatial logic for neural repair across distinct pathological contexts.

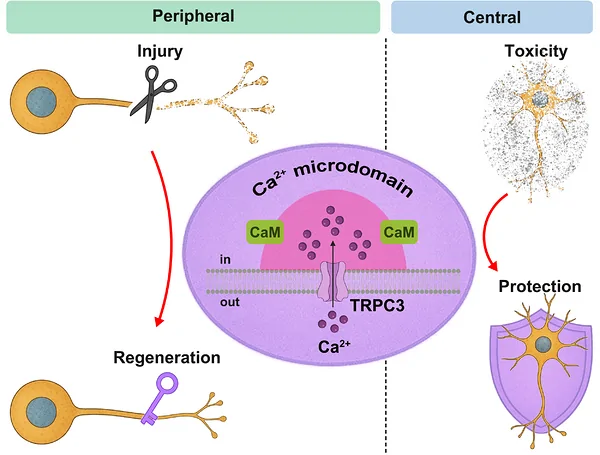

## 1 Introduction

The adult mammalian nervous system exhibits a markedly restricted capacity for structural and functional recovery following injury or neurodegeneration [1, 2, 3, 4, 5]. A central obstacle in overcoming this limitation is the “calcium paradox”: while Ca^2+^ signaling is indispensable for neuronal plasticity and growth [3], global Ca^2+^ elevations—often triggered by pathological insults—frequently culminate in Ca^2+^ overload and neurotoxicity [6], rather than repair. Consequently, the field has struggled to reconcile the dual nature of Ca^2+^ as both a driver of neuronal death and an essential messenger for repair. Emerging evidence suggests that the instructional specificity required to govern repair programs may reside not in the magnitude of global fluxes, but in the spatiotemporal precision of discrete signaling events [7, 8]. However, whether a sub‐cellular Ca^2+^ signaling modality exists that can bypass global toxicity to instruct repair fates—and, if so, whether such a mechanism might also operate in distinct neural contexts—remains unknown.

Ca^2+^ microdomains [8, 9, 10] represent a critical yet poorly resolved signaling modality capable of encoding spatially confined information, but the molecular identity, functional logic, and in vivo significance of spontaneous near‐membrane microdomain Ca^2+^ hotspots [11, 12] remain elusive. While these hotspots have been partially attributed to TRPA1‐mediated Ca^2+^ influx in specific sensory subsets [12], this mechanism cannot explain their broad occurrence across heterogeneous neuronal populations [13] or their diverse physiological outputs. A critical unresolved question, therefore, is whether a common molecular basis underlies these spontaneous Ca^2+^ microdomains, enabling them to couple extracellular Ca^2+^ entry to repair‐permissive intracellular signaling.

Here we identify TRPC3 [14, 15] as a driver of spontaneous near‐membrane Ca^2+^ hotspots that instruct an intrinsic pro‐repair program. We show that TRPC3‐mediated Ca^2+^ microdomains are necessary and sufficient for axonal regrowth in cultured DRG neurons and essential for peripheral nerve regeneration and functional recovery in vivo. Extending this mechanism to the central nervous system, our data further suggest that TRPC3 gain‐of‐function is associated with enhanced dopaminergic neuron survival and motor function in a Parkinson's disease model, and that this pro‐repair phenotype is observed in human ESC‐derived dopaminergic neurons. Together, our findings identify TRPC3 as a molecular driver of Ca^2+^ microdomains that in turn function as a signaling module for peripheral axon regeneration, with implications for neuroprotection in the central nervous system. These results may uncover a critical mechanism of endogenous neural repair and position TRPC3 as a potential molecular target for therapeutic strategies against nerve injury and neurodegenerative disorders.

## 2 Results

### 2.1 TRPC3 Mediates Spontaneous Microdomain Ca^2+^ Hotspots in Sensory Neurons

**FIGURE 1:**
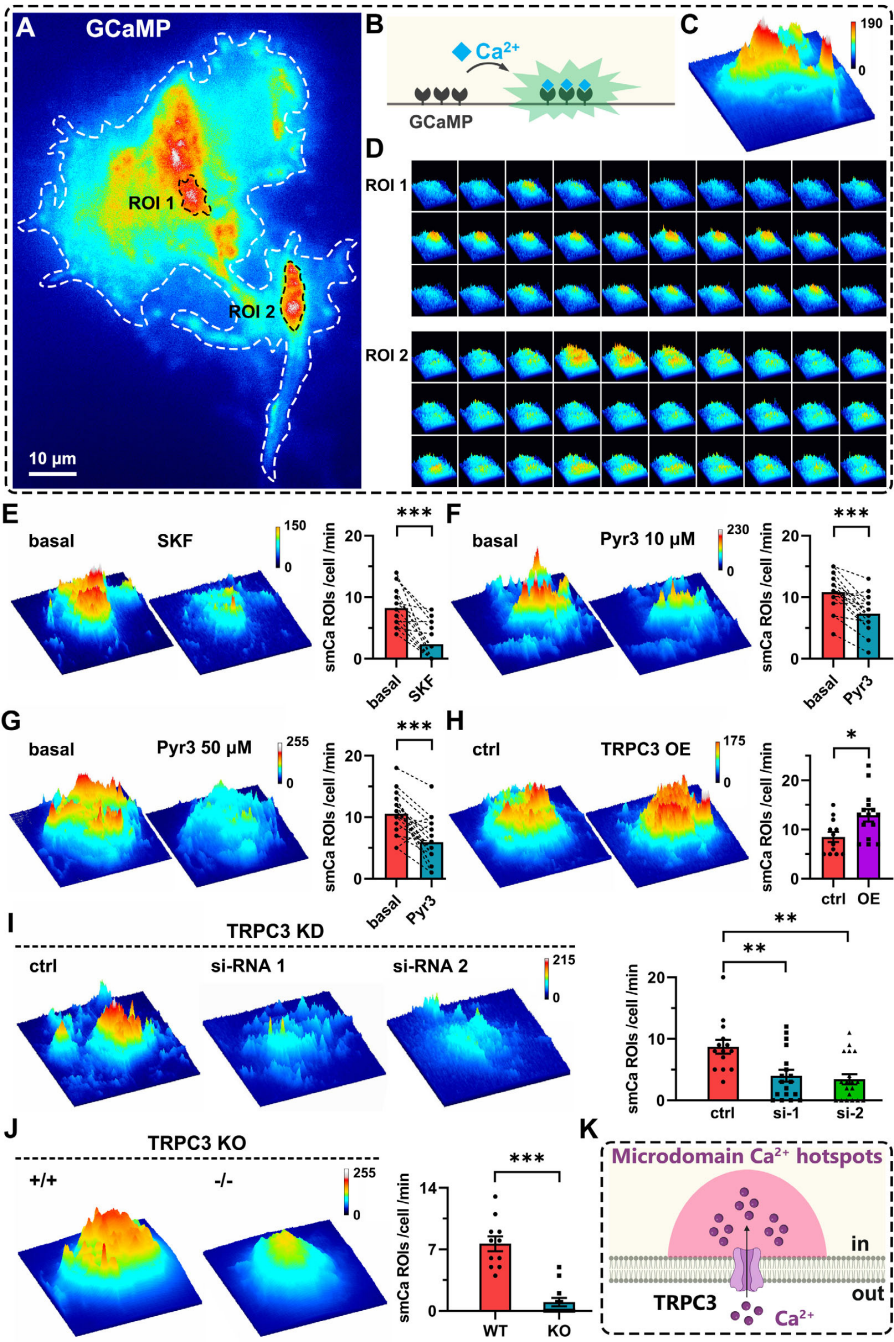
TRPC3 mediates spontaneous microdomain Ca^2+^ hotspots (smCa) in sensory DRG neurons. (A) TIRF image of a 20‐h cultured rat dorsal root ganglion (DRG) neuron transfected with the membrane‐anchored Ca^2+^ indicator Lck‐GCaMP5g. (B) Schematic depicting membrane‐localized Lck‐GCaMP5g (black) binding Ca^2+^ and emitting green fluorescence. (C) Whole‐cell surface plot of membrane‐proximal Ca^2+^ signals in the neuron shown in (A). (D) Representative 30‐s montages of membrane‐proximal Ca^2+^ hotspots from two regions of interest (ROIs) in (A). Montages are arranged left to right and top to bottom. (E) Whole‐cell surface plot (left) and quantification of the number of active ROIs per cell per minute (right) before and after treatment with 100 µm SKF‐96365 (a non‐selective TRPC antagonist). *n* = 17 neurons. (F, G) Whole‐cell surface plots (left) and quantification of active ROIs per cell per minute (right) before and after treatment with 10 or 50 µm Pyr3 (a selective TRPC3 antagonist). *n* = 18 neurons for (F), 22 neurons for (G). (H) Whole‐cell surface plots (left) and quantification (right) in control (Lck‐GCaMP5g) and TRPC3‐overexpression (Lck‐GCaMP5g + TRPC3) groups. *n* = 12 neurons for ctrl, 15 neurons for OE. (I) Whole‐cell surface plots (left) and quantification (right) in control (Lck‐GCaMP5g + scramble siRNA) and TRPC3‐knockdown (Lck‐GCaMP5g + TRPC3‐siRNA) groups. *n* = 14 neurons for ctrl, 18 neurons for siRNA #1, 19 neurons for siRNA #2. (J) Whole‐cell surface plots (left) and quantification (right) in WT and TRPC3^−^/^−^ mice. *n* = 11 neurons for WT, 13 neurons for KO. (K) Schematic illustrating spontaneous TRPC3‐mediated extracellular Ca^2+^ influx giving rise to membrane‐proximal microdomain Ca^2+^ hotspots. Data are presented as mean ± S.E.M., with all data points shown. Each data point represents one neuron. Statistical tests: Wilcoxon matched‐pairs signed‐rank test (E); paired Student's *t*‐test (F, G); Student's *t*‐test (H, J); one‐way ANOVA (I). *p < 0.05; **p < 0.01; ***p < 0.001.

We previously [12] identified spontaneous near‐membrane Ca^2+^ microdomain (smCa) hotspots as a characteristic signaling feature of primary sensory neurons, broadly distributed across the soma, axon initial segments, and growth cones (Figure 1A–D, Figure S1 and Movies S1 and S2). While our earlier work implicated the TRPA1 channel in generating a subset of these signals, we observed that pharmacological inhibition or genetic ablation of TRPA1 eliminated only a fraction of total smCa activity, suggesting the existence of a more dominant molecular generator. Notably, whereas TRPA1 is primarily restricted to specialized nociceptive neurons [13], we found that smCa hotspots are a near‐universal feature across heterogeneous DRG neuronal subtypes. Consistent with this, we found that ruthenium red—a broad‐spectrum TRP channel antagonist—almost completely abolished smCa activity across all populations [12]. These observations led us to hypothesize that a more widely expressed TRP channel serves as the primary driver of the smCa‐mediated signaling axis.

To identify this channel, we first tested the non‐selective TRPC inhibitor SKF‐96365 (SKF), which markedly diminished smCa activity (Figure 1E). Among the seven TRPC family members [16], RNA‐seq analysis revealed that TRPC3 is the most abundantly expressed in 20‐hour cultured DRG neurons (Figure S2). Consistent with this, the selective TRPC3 antagonist Pyr3 [17] significantly reduced smCa hotspots in a dose‐dependent manner (Figure 1F,G), supporting a functional role for TRPC3 in mediating smCa events. Gain‐ and loss‐of‐function approaches further validated this conclusion: TRPC3 overexpression robustly enhanced smCa activity, whereas TRPC3 knockdown or genetic ablation markedly reduced it (Figure 1H–J, Figures S3 and S4). Importantly, reintroduction of siRNA‑resistant TRPC3 fully restored smCa activity in TRPC3‑knockdown neurons, whereas a pore‑dead (Ca^2+^ impermeable) mutant [18, 19] failed to do so (Figure S7), confirming that the channel's ion‑conducting function is required. Together, these results demonstrate that TRPC3 is a key mediator of spontaneous smCa hotspots in sensory neurons (Figure 1K).

### 2.2 TRPC3 and smCa Contribute to Sensory Nerve Regeneration

**FIGURE 2:**
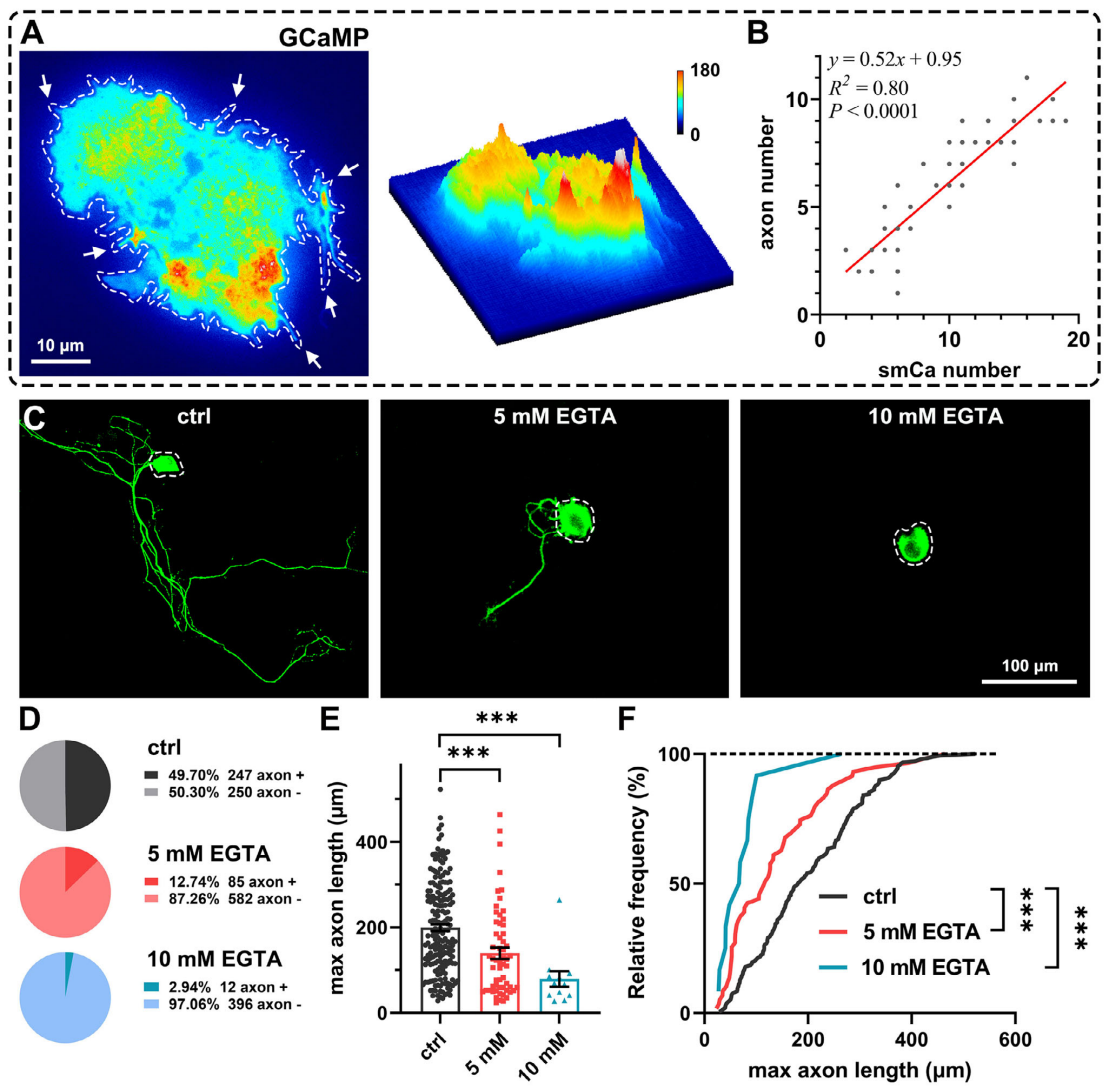
Sensory nerve regeneration correlates with smCa and extracellular Ca^2+^. (A) TIRF image of a 20‐h cultured rat DRG neuron transfected with Lck‐GCaMP5g (left). Arrows indicate regenerated axons. The corresponding whole‐cell surface plot of membrane‐proximal Ca^2+^ signals is shown on the right. (B) Correlation analysis between the number of regenerated axons and the number of smCa ROIs. (C) Confocal images of 24‐h cultured sensory DRG neurons grown in control medium, medium supplemented with 5 mm EGTA, or medium supplemented with 10 mm EGTA. (D) Percentage of regenerated‐axon–positive cells in the control, 5 mm EGTA, and 10 mm EGTA groups shown in (C). (E) Quantification of the maximum regenerated‐axon length per cell in the groups shown in (C). (F) Relative frequency distribution of maximum regenerated‐axon length per cell in the control, 5 mm EGTA, and 10 mm EGTA groups. Data are presented as mean ± S.E.M., with all data points shown. Each data point represents one neuron and the *n* value is shown in (D) panel. Statistical tests: one‐way ANOVA. ***p < 0.001.

During DRG neuron culture and smCa imaging, we observed a positive correlation between the number of regenerated axons and smCa activity (Figure 2A,B), suggesting that smCa contributes to axon regeneration under physiological conditions. Given the complete dependence of smCa signals on extracellular Ca^2+^ influx [12], we therefore assessed its necessity for axon regeneration by removing extracellular Ca^2+^. Chelating Ca^2+^ in the culture medium with EGTA markedly reduced both the percentage of axon‐regenerating neurons and the maximum regenerated‐axon length in a dose‐dependent manner (Figure 2C–F). It should be noted that EGTA chelates all extracellular Ca^2+^ and may therefore influence broader Ca^2+^‑dependent events beyond smCa signaling. To further determine how extracellular Ca^2+^ influences regeneration, we screened Ca^2+^ channel types. Broad inhibition of TRP channels with ruthenium red profoundly impaired axon regeneration (Figure S5), while inhibition of voltage‐gated calcium channels showed no effect, implying TRP channel activity is likely required for axon regeneration.

**FIGURE 3:**
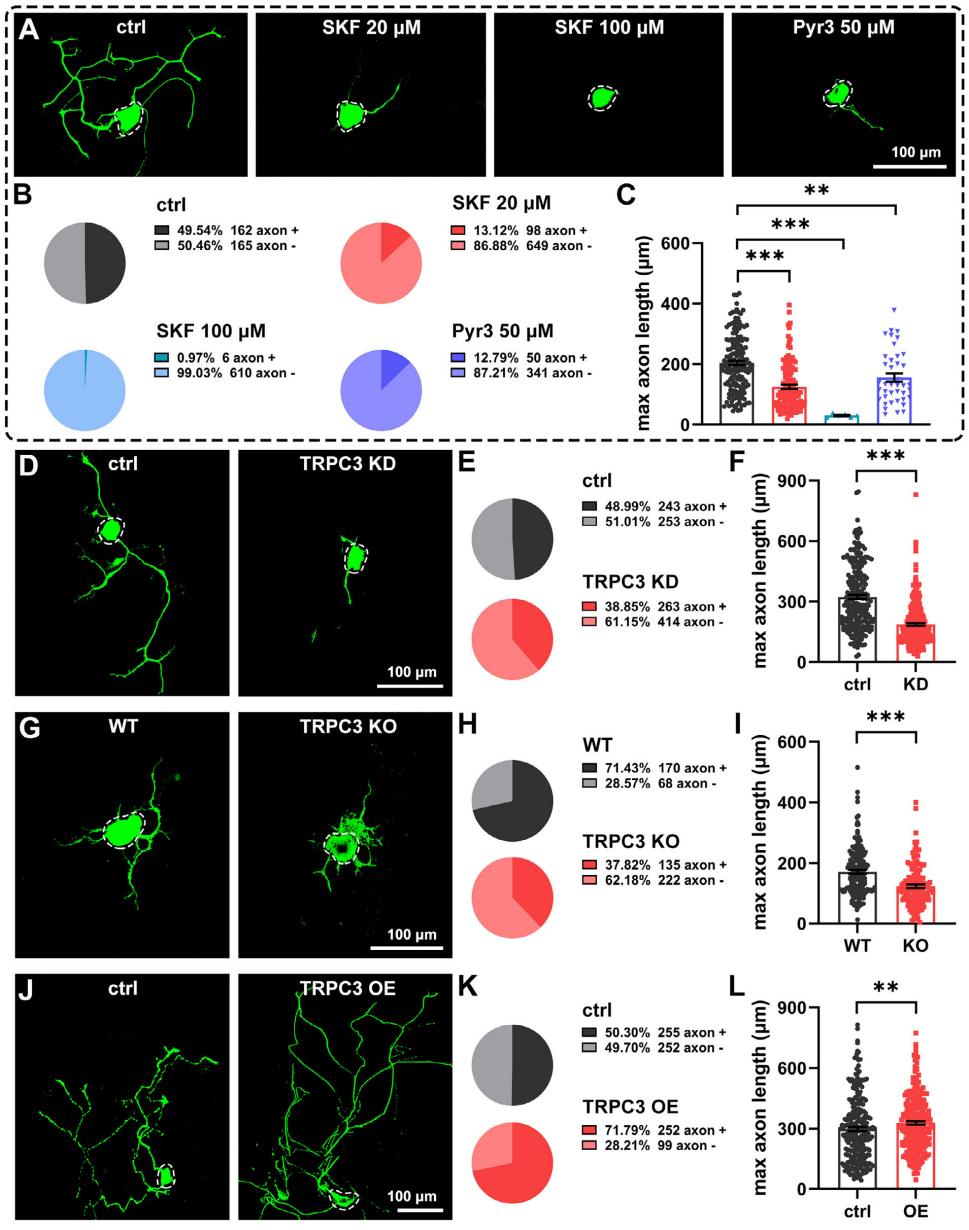
Sensory nerve regeneration depends on TRPC3. (A) Confocal images of 24‐h cultured rat sensory DRG neurons maintained in control medium or medium supplemented with 20 µm SKF‐96365, 100 µm SKF‐96365, or 50 µm Pyr3. (B) Percentage of regenerated‐axon‐positive cells in the control, 20 µm SKF, 100 µm SKF, and 50 µm Pyr3 groups shown in (A). (C) Quantification of maximum regenerated‐axon length per cell in the groups shown in (A). (D) Confocal images of 36‐h cultured rat sensory DRG neurons in control (mCherry + scramble siRNA) and TRPC3‐knockdown (mCherry + TRPC3‐siRNA) groups. (E) Percentage of regenerated‐axon‐positive cells in the control and TRPC3‐knockdown groups shown in (D). (F) Quantification of maximum regenerated‐axon length per cell in the groups shown in (D). (G–I) Same analyses as in (D–F), but comparing 24‐h cultured mouse sensory DRG neurons in WT and TRPC3^−^/^−^ groups. (J–L) Same analyses as in (D–F), but comparing control (mCherry) and TRPC3‐overexpression (mCherry + TRPC3) groups. Data are presented as mean ± S.E.M., with all data points shown. Each data point represents one neuron and the n value is shown in the percentage panel. Statistical tests: one‐way ANOVA (C); Mann–Whitney test (F, I, L). **p < 0.01; ***p < 0.001.

Given that smCa activity is mediated by TRPC3 (Figure 1) and correlates positively with axon regeneration (Figure 2A,B), we hypothesized that TRPC3 contributes to regenerative capacity. Consistent with this idea, pharmacological inhibition of TRPC3 using either the non‐selective antagonist SKF or the TRPC3‐selective antagonist Pyr3 markedly reduced both the percentage of regenerated‐axon‐positive neurons and the maximum regenerated‐axon length in a dose‐dependent manner (Figure 3A–C). Likewise, TRPC3 knockdown—but not knockdown of other TRPC family members—significantly impaired axon regeneration (Figure 3D–F, Figure S6A,B). Additionally, the effect of TRPC3 knockdown on axon regeneration became more pronounced with prolonged culture time (Figure S6C). TRPC3 knockout produced similar deficits (Figure 3G–I). In contrast, TRPC3 overexpression enhanced regenerative ability, increasing both the proportion of regenerating neurons and axon length (Figure 3J–L). Moreover, expression of siRNA‑resistant TRPC3 fully rescued axonal outgrowth in knockdown neurons, whereas a pore‑dead mutant failed to do so (Figure S7), indicating that the ion‑conducting function of TRPC3 is essential for its pro‑regenerative effect.

### 2.3 Calmodulin is Involved in TRPC3‐smCa Mediated Sensory Nerve Regeneration

**FIGURE 4:**
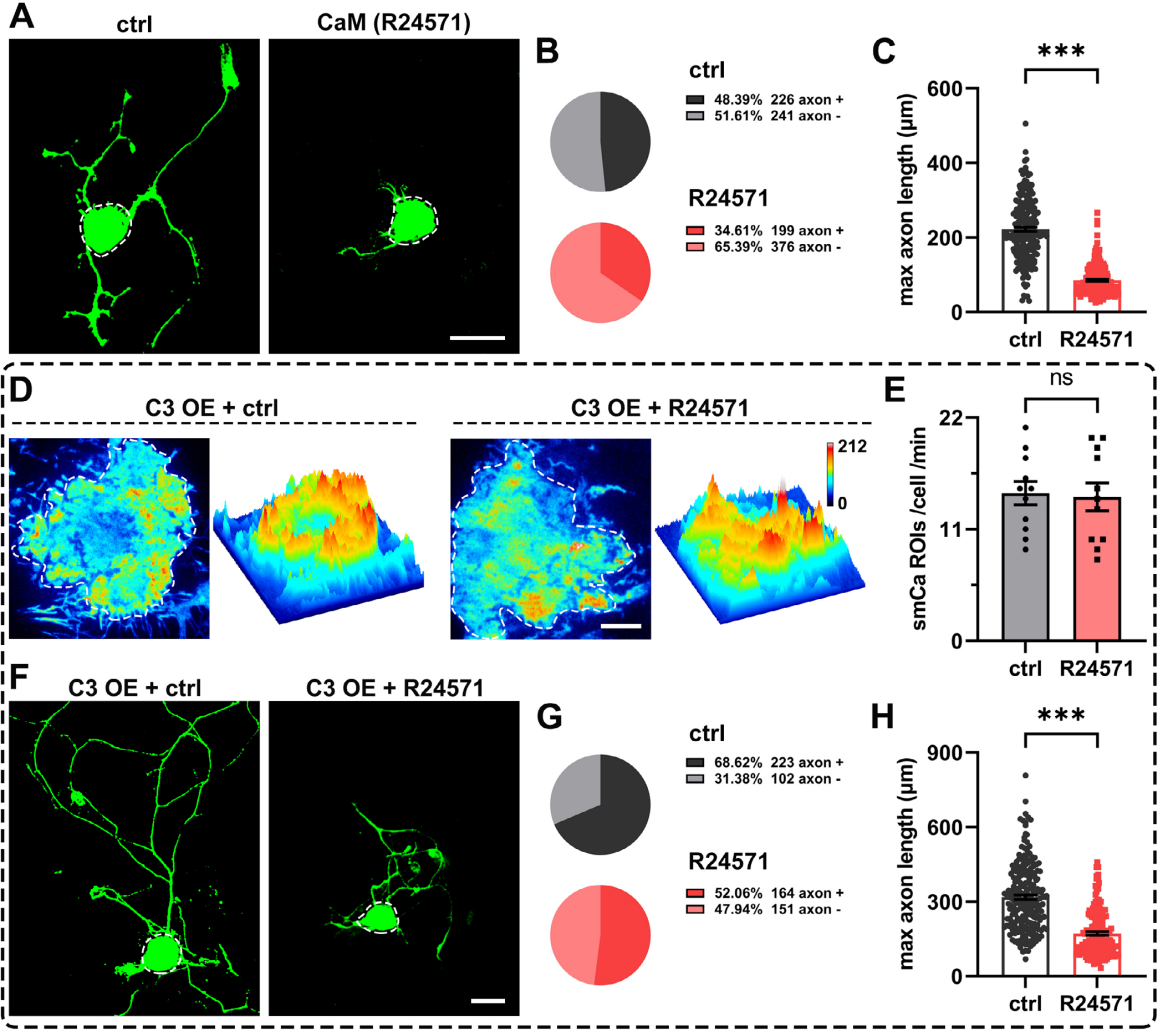
Sensory nerve regeneration depends on calmodulin. (A) Confocal images of 24‐h cultured rat sensory DRG neurons in control medium or medium containing 0.8 µm R24571 [a selective calmodulin (CaM) antagonist]. Scale bar, 50 µm. The complete comparison across all treated groups (CaN, CaMKK, and CaMKII inhibitors) is provided in Figure S8. (B) Percentage of regenerated‐axon‐positive cells in the control and R24571 groups shown in (A). (C) Quantification of maximum regenerated‐axon length per cell in the groups shown in (A). (D, E) Whole‐cell surface plot (left) and quantification of active smCa ROI number per cell per minute (right) in rat DRG neurons overexpressing TRPC3, with or without R24571 treatment. Scale bar, 10 µm. *n* = 11 neurons for ctrl and R24571. (F–H) Same analyses as in (A–C), but in rat DRG neurons overexpressing TRPC3 for 36 h, with or without R24571 treatment. Scale bar, 50 µm. Data are presented as mean ± S.E.M., with all data points shown. Each data point represents one neuron and the *n* value is shown in the percentage panel. Statistical tests: Student's *t*‐test. ns, not significant; ***p < 0.001.

To further elucidate how TRPC3‐mediated smCa activity (Figure 1) couples to its pro‐regenerative function (Figure 3), we examined calmodulin (CaM), a Ca^2+^‐binding protein known to play pivotal roles in axon growth and regeneration [20, 21]. Pharmacological inhibition of CaM with its selective antagonist R24571 markedly reduced regenerative capacity (Figure 4A–C), suggesting that CaM may serve as an essential Ca^2+^ sensor linking TRPC3‐driven smCa signals to axon regeneration. To determine the hierarchical relationship between smCa and CaM, we treated TRPC3‐overexpressing neurons with R24571. Notably, CaM blockade had no significant effect on smCa activity but dramatically impaired axon regeneration (Figure 4D–H), indicating that CaM functions downstream of smCa to promote axonal outgrowth. We next screened for downstream CaM effectors, including calcineurin (CaN), CaMKK, and CaMKII. Our preliminary results show that CaN inhibition robustly suppressed axon regeneration, whereas blocking CaMKK or CaMKII had no appreciable effect (Figure S8), suggesting CaN as a potential downstream mediator of CaM in this regenerative process.

### 2.4 TRPC3 Promotes Nerve Regeneration and Sensory Recovery In Vivo

**FIGURE 5:**
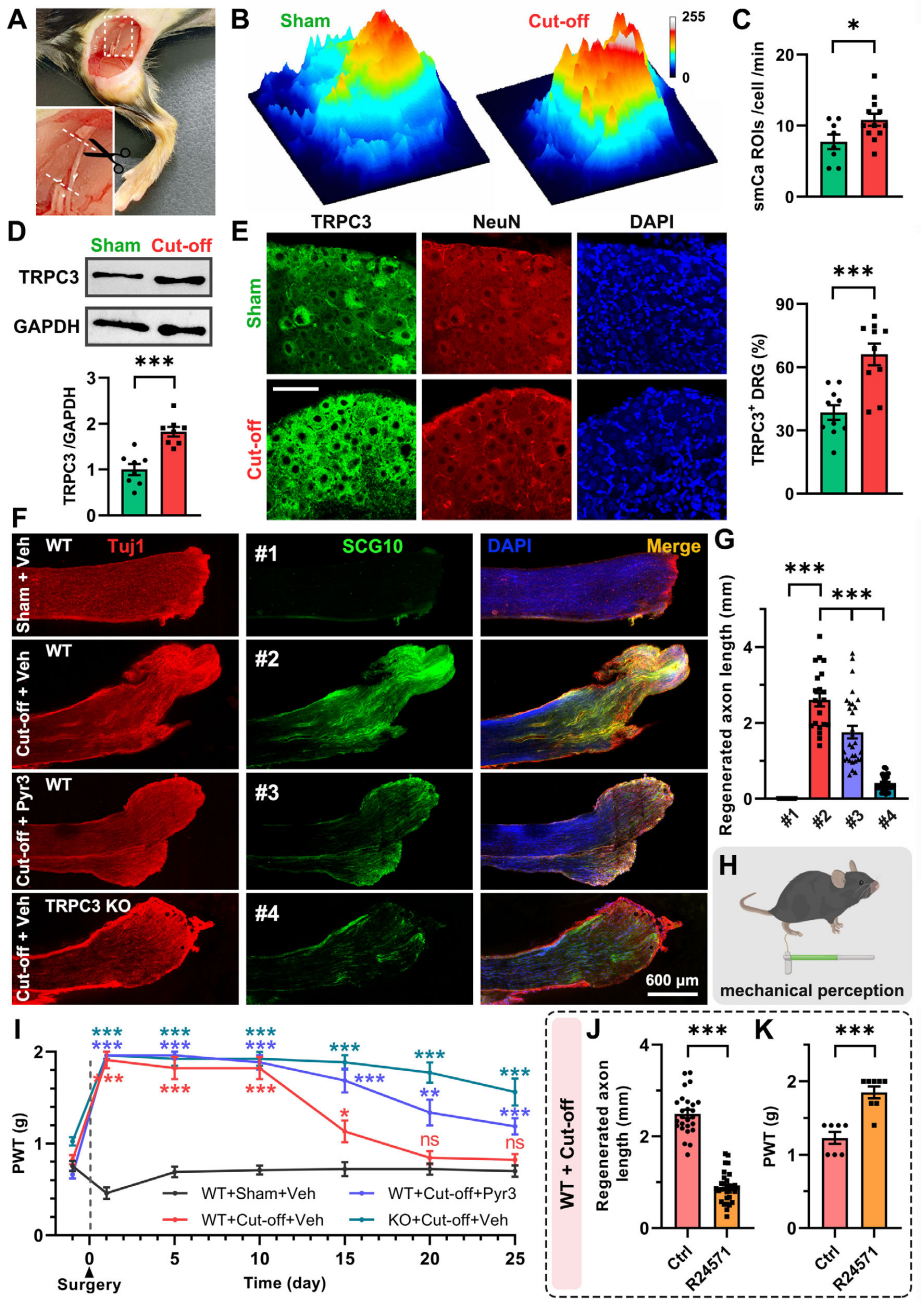
TRPC3 regulates sensory nerve regeneration and mechanical perception recovery in vivo. (A) Anatomical photograph illustrating the sciatic nerve cut‐off model. (B) Whole‐cell surface plots of membrane‐proximal Ca^2+^ signals in sham‐operated and sciatic nerve cut‐off adult male C57 mice, obtained 4 days after surgery. (C) Quantification of total smCa ROIs per cell per minute in the sham and cut‐off groups. Each data point represents one neuron. *n* = 8 for sham, 12 for cut‐off. (D) Western blot analysis of TRPC3 and loading control GAPDH in sham and cut‐off groups, collected 4 days after surgery (Top). Quantification of TRPC3 protein levels normalized to GAPDH (Bottom). Each data point represents one mouse. *n* = 8 per group. (E) Confocal images of TRPC3 immunofluorescence in sham and cut‐off groups, collected 4 days after surgery (Left). Proportion of TRPC3‐positive DRG neurons, quantified from multiple slices (Right). Each data point represents one DRG slice. *n* = 10 per group. Scale bar, 100 µm. (F) Confocal images showing existing axons (Tuj1, red) and newly regenerated axons (SCG10, green) in WT + sham + vehicle, WT + cut‐off + vehicle, WT + cut‐off + Pyr3 (1 mm, 100 µL; intramuscularly injected into the surgical site daily after surgery), and TRPC3^−^/^−^ + cut‐off + vehicle groups, collected 4 days post‐surgery. (G) Quantification of newly regenerated axon length in the groups described in (F). Each data point represents one regenerated fiber. *n* = 18, 27, 36, 47 for each group, respectively. (H) Schematic illustrating the assessment of mechanical perception in mice. (I) Mechanical sensory threshold measurements in WT + sham + vehicle, WT + cut‐off + vehicle, WT + cut‐off + Pyr3, and TRPC3^−^/^−^ + cut‐off + vehicle groups before and after sciatic nerve cut‐off surgery. *n* = 9, 9, 8, 8 mice for each group, respectively. (J) Quantification of newly regenerated axon length in the groups of WT + cut‐off + vehicle and WT + cut‐off + R24571 (20 µm, 100 µL; intramuscularly injected into the surgical site daily after surgery), collected 4 days post‐surgery. Each data point represents one regenerated fiber. *n* = 26 for ctrl, 31 for R24571. (K) Mechanical sensory threshold measurements in WT + cut‐off + vehicle and WT + cut‐off + R24571 groups, collected 15 days post‐surgery. Each data point represents one mouse. *n* = 7 for ctrl, 8 for R24571. Data are presented as mean ± S.E.M., with all data points shown. Statistical tests: Student's *t*‐test (C, D, E, J, K); one‐way ANOVA (G, I). ns, not significant; *p < 0.05; **p < 0.01; ***p < 0.001.

To determine whether TRPC3 contributes to nerve regeneration in vivo, we employed a sciatic nerve cut‐off model (Figure 5A). We observed a marked increase in spontaneous microdomain Ca^2+^ signals (Figure 5B,C), and in the protein level and proportion of TRPC3‐positive DRG neurons (Figure 5D,E) following nerve transection, indicating that TRPC3‐mediated smCa activity may be engaged during injury and subsequent self‐repair. We further assessed axonal regeneration by quantifying SCG10‐positive fibers, which specifically mark newly regenerated axons [22]. Robust axon regrowth was observed in the nerve cut‐off group, whereas treatment with the TRPC3‐selective antagonist Pyr3 or genetic deletion of TRPC3 markedly impaired regeneration (Figure 5F,G). Furthermore, when TRPC3 was reintroduced via viral overexpression in TRPC3‑knockout DRG neurons, sciatic nerve regeneration was dramatically enhanced (Figure S9), indicating that TRPC3 is required for axon regeneration in vivo.

Sensory DRG neurons and sciatic nerves are essential for mechanical perception and thus critical for mammalian survival [23]. Consistent with previous observations, we found that mechanical sensitivity was abolished immediately after sciatic nerve transection and gradually recovered over the course of ∼20 days of spontaneous healing (Figure 5H,I). Notably, inhibition of TRPC3 or CaM significantly delayed both neural repair and the recovery of mechanical sensation (Figure 5F–K, Figure S10). Together, these findings show that TRPC3 drives spontaneous microdomain Ca^2+^ activity and promotes axon regeneration, thereby providing a mechanism that supports peripheral sensory recovery in vivo.

### 2.5 TRPC3 Gain‐of‐Function in the SNc Improves Motor Function in a PD Model

**FIGURE 6:**
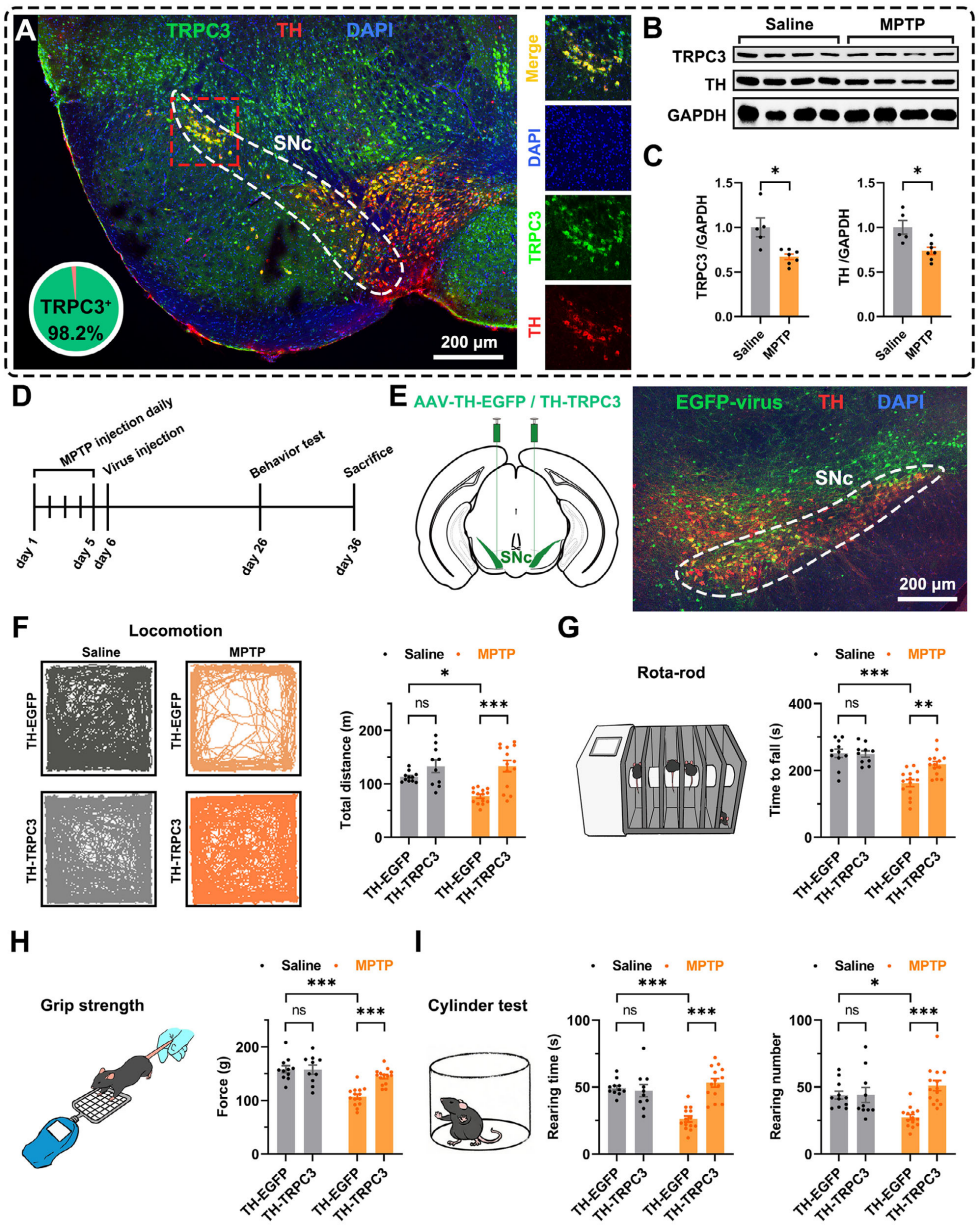
TRPC3 overexpression restores motor function in PD mouse model. (A) Immunofluorescence (IF) images of the substantia nigra pars compacta (SNc) in adult C57 mice showing TRPC3‐positive cells among TH‐positive neurons, with quantification indicated. (B) Representative western blot bands for TRPC3, TH, and GAPDH from saline‑ and MPTP‑treated mice at 21 days post‑injection. (C) Quantification of TRPC3 and TH protein levels in saline and MPTP groups. *n* = 5 for saline, 7 for MPTP. (D) Schematic outline of the experimental timeline, including MPTP administration, viral injection, and behavioral testing. (E) Brain atlas indicating the stereotaxic coordinates for bilateral viral injection into the SNc (left), and IF images showing viral infection efficiency (right). (F) Trajectory plots (left) and total‐distance data (right) for the ctrl virus + saline, TRPC3 OE virus + saline, ctrl virus + MPTP, and TRPC3 OE virus + MPTP groups. n = 11, 10, 14, 14 mice for each group, respectively. (G) Schematic of the rota‐rod assay (left) and quantification of time to fall (right) for the groups described in (F). (H) Schematic of the grip‐strength test (left) and quantification of gripping force (right) for the groups in (F). (I) Schematic of the cylinder test (left) and quantification of rearing time and rearing counts (right) for the groups in (F). Data are presented as mean ± S.E.M., with all data points shown. Each data point represents one mouse. Statistical tests: Student's *t*‐test (C); two‐way ANOVA (F–I). ns, not significant; *p < 0.05; **p < 0.01; ***p < 0.001.

In Parkinson's disease (PD), the midbrain dopaminergic system is characterized by progressive neuronal loss (soma and axons). Accordingly, therapeutic approaches that delay neuronal loss or enhance axonal regeneration may have major translational potential. We next asked whether this TRPC3‐mediated regenerative mechanism extends to the central nervous system, focusing on the substantia nigra pars compacta (SNc) given its critical relevance to PD [24, 25]. TRPC3 was readily detected in the SNc and exhibited extensive colocalization with tyrosine hydroxylase (TH), a dopaminergic (DA) neuron marker (98.2%; Figure 6A), implying a potential role for TRPC3 in DA neuron function. Additionally, in the 21 d MPTP‐induced PD mouse model [26], TRPC3 protein levels were markedly reduced (Figure 6B,C), accompanied by a pronounced decrease in TH expression.

To test whether restoring TRPC3 expression could mitigate PD‐related motor deficits, we bilaterally delivered an AAV carrying TRPC3 with TH promoter into the SNc. The experimental workflow and viral infection efficiency are shown in Figure 6D,E and Figure S11. Across a battery of behavioral assays—including open‐field locomotion, rotarod performance, grip strength, and cylinder testing—TRPC3 overexpression significantly improved motor function relative to control virus–treated mice (Figure 6F–I). Together, these results suggest that TRPC3 gain‐of‐function is associated with functional benefits in a PD context, pointing to TRPC3 as a promising therapeutic target for Parkinson's disease.

### 2.6 TRPC3 Gain‐of‐Function Confers Neuroprotection in a PD Model

**FIGURE 7:**
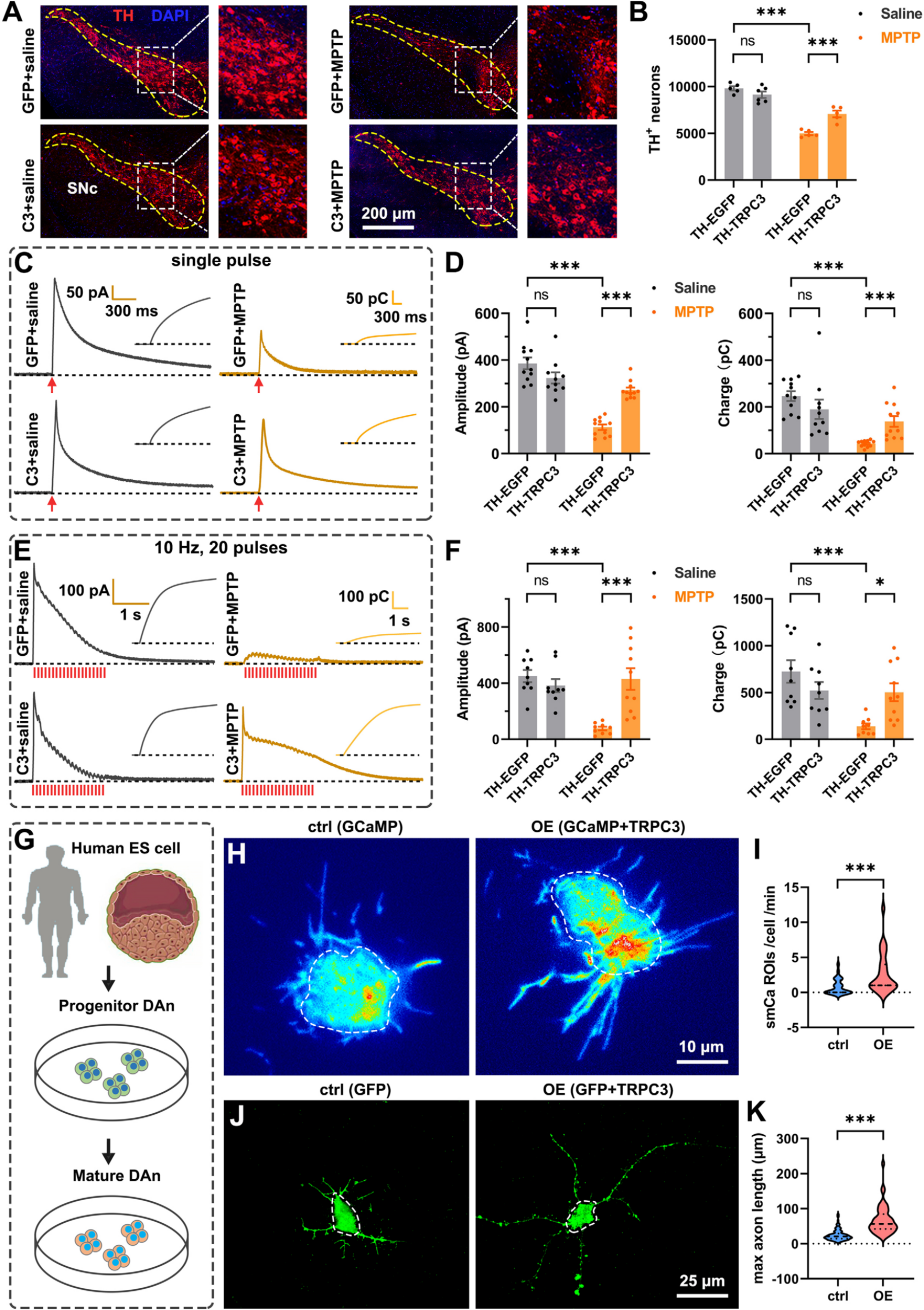
TRPC3 overexpression is associated with dopaminergic neuron protection in a Parkinson's disease model and enhances smCa activity and axon regeneration in human ESC‐derived DA neurons. (A) Representative immunofluorescence images of the substantia nigra pars compacta (SNc) from the following experimental groups: control virus + saline, TRPC3‐overexpressing (OE) virus + saline, control virus + MPTP, and TRPC3 OE virus + MPTP. (B) Quantitative analysis of tyrosine hydroxylase‐positive (TH^+^) neurons in the groups shown in (A). Each data point represents one mouse. *n* = 5, 6, 5, 5 for each group, respectively. (C) Representative electrochemical traces recorded with a carbon‐fiber electrode following single‐pulse electrical stimulation, showing amplitude (main panel) and charge transfer (inset). (D) Summary data of signal amplitude and charge for the recordings presented in (C). Each data point represents one brain slice. n = 11, 10, 11, 11 for each group, respectively. (E, F) Corresponding representative traces (E) and quantitative analysis (F) under burst stimulation (10 Hz, 20 pulses). Each data point represents one brain slice. *n* = 9, 9, 9, 10 for each group, respectively. (G) Schematic illustration of the differentiation protocol for obtaining dopaminergic (DA) neurons from human embryonic stem (ES) cell lines. (H, I) TIRF imaging of Ca^2+^ microdomains in mature DA neurons (H) and the corresponding quantification (I). *n* = 28 cells for ctrl, 27 cells for OE. (J, K) Confocal microscopy images of regenerated axons (J) and their quantitative assessment (K). *n* = 123 cells for ctrl, 99 cells for OE. Data are presented as mean ± S.E.M., with all data points shown, or as violin plots with median and quartiles. Statistical tests: Student's *t*‐test (I, K); two‐way ANOVA (B, D, F). ns, not significant; *p < 0.05; ***p < 0.001.

To assess whether TRPC3 gain‐of‐function confers protection in the PD model, we first quantified dopaminergic (DA) neuron survival in the SNc. TRPC3 overexpression was associated with increased survival of DA neurons following MPTP treatment compared with the control virus‐treated group (Figure 7A,B). Additionally, we found that in situ CaM blockade dramatically decreased the survival of DA neurons and impaired the motor behaviors (Figure S12). We next assessed dopamine release using amperometric carbon‐fiber recordings and found that both the peak amplitude and total charge evoked by either single‐pulse or burst stimulation were substantially enhanced in TRPC3‐overexpressing mice after MPTP administration (Figure 7C–F). These findings suggest that TRPC3 gain‐of‐function is correlated with enhanced DA neuron viability and improved neurotransmission capacity in a PD pathological context.

To further explore the translational relevance of our findings, we generated human embryonic stem cell lines (hESC)–derived dopaminergic neurons (Figure S13) [27]. TRPC3 overexpression in hESC‐derived DA neurons significantly increased the number of spontaneous microdomain Ca^2+^ (smCa) hotspots and robustly promoted axonal regeneration (Figure 7G–K), similar to the effects observed in peripheral sensory DRG neurons. These results are consistent with the TRPC3‐mediated enhancement of spontaneous smCa activity and axon regeneration observed in SH‐SY5Y cells (Figure S14). Together with our observations in the PD mouse model, these findings suggest that TRPC3‐mediated Ca^2+^ microdomains may represent a pro‐repair mechanism that operates across different neuronal contexts.

**FIGURE 8:**
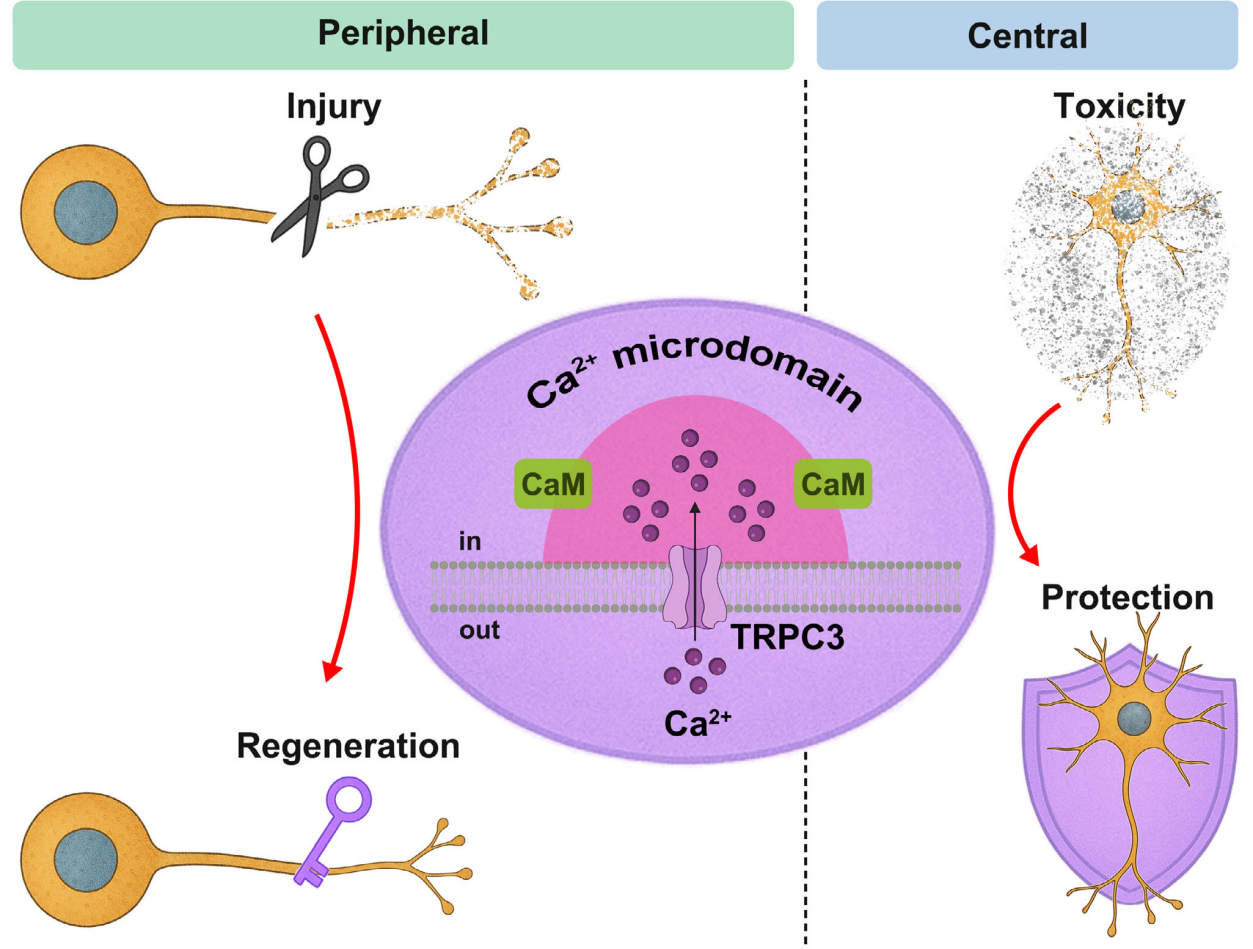
TRPC3 drives peripheral nerve regeneration via localized calcium microdomains, with correlative neuroprotection in the central nervous system. Schematic model illustrating how TRPC3 promotes neural repair through the generation of spontaneous Ca^2+^ microdomains. Left: Following peripheral nerve injury, sensory neurons engage TRPC3‐dependent Ca^2+^ microdomains, which act as a molecular trigger for the intrinsic axon regeneration program via calmodulin pathway, driving robust elongation and functional recovery. Right: In the central nervous system, dopaminergic neurons challenged with MPTP undergo degeneration; TRPC3‐mediated Ca^2+^ microdomains are associated with neuronal survival and sustained dopamine release. Together, this model identifies TRPC3 as a molecular driver of spatially confined Ca^2+^ signals that promote regeneration in the PNS and correlate with neuroprotection in the CNS, positioning TRPC3 as a potential therapeutic target.

In summary, our findings identify TRPC3‐driven Ca^2+^ microdomains as an instructive module for peripheral nerve regeneration, with implications for neuroprotection in the central nervous system (Figure 8). This work offers insight into the long‐standing calcium paradox by revealing that neural repair can be governed by sub‐cellular spatial precision rather than global signal magnitude. Our results suggest a spatial logic of endogenous repair and point to TRPC3 as a potential therapeutic target for nerve injury and neurodegenerative disorders.

## 3 Discussion

The restoration of structural and functional integrity following neural insult remains a formidable challenge in regenerative medicine [28]. Our study identifies TRPC3 as a molecular driver of Ca^2+^ microdomains that in turn function as a signaling module for peripheral nerve regeneration, with implications for neuroprotection in the central nervous system (Figure 8). These findings add to the emerging view that finely compartmentalized Ca^2+^ dynamics—rather than global Ca^2+^ elevations—constitute instructive signals for neural repair. They further highlight TRPC3 as a potential molecular entry point for therapeutic modulation in nerve injury and neurodegenerative disorders.

### 3.1 TRPC3‑smCa as a Localized Repair Module for Axon Regeneration in the PNS

Ca^2+^ signaling faces a specificity problem: how can one messenger trigger distinct responses? The solution may lie in Ca^2+^ microdomains—localized high‑Ca^2+^ regions near open channels that act as the elementary building blocks of signaling [29, 30, 31]. These microdomains are versatile, generating events such as Ca^2+^ sparks and sparklets that can summate into global transients or act locally [32, 33]. In astrocytes, microdomain Ca^2+^ regulates neuronal activity and repair [11, 34]. In vascular smooth muscle, global vs. subsurface Ca^2+^ signals paradoxically control constriction vs. relaxation [32]. Thus, microdomain Ca^2+^ may reveal how spatial organization ensures signaling specificity and versatility [32].

The discovery that TRPC3 generates smCa hotspots complements earlier work [12] showing that TRPA1 accounts for only partial of these events, despite their presence across neurons of all diameters [13, 35]. The present data reveal that TRPC3 is the predominant TRPC isoform in cultured DRG neurons (Figure S2) and that manipulating its activity—pharmacologically or genetically—modulates smCa signals bidirectionally (Figure 1, Figure S7). Although both TRPA1 and TRPC3 contribute to smCa, their downstream roles appear distinct: TRPA1‐mediated smCa regulates spontaneous neurotransmitter release through cAMP‐PKA signaling pathway [12], whereas TRPC3‐driven hotspots promote axon regeneration (Figure 3, Figures S7 and S15) through calmodulin‐dependent mechanisms (Figure 4, Figure S10). The spatial organization, amplitude, and kinetics of channel‐specific microdomains may thus underlie divergent signaling outputs [36]. However, the precise nanodomain architecture and downstream molecular cascade [37] remain unresolved and will require future imaging and biochemical dissection.

TRPC channels play essential roles in axonal regeneration and guidance. TRPC1 assembles mechanosensitive channels on growth cones to regulate axon outgrowth direction and extension [38], and is also required for chemoattractant‐induced turning and midline guidance [39]. TRPC3 mediates BDNF‐dependent growth cone steering [40]. TRPC4 is increased after nerve injury and promotes neurite outgrowth [41]. Collectively, these studies suggest that TRPC‐dependent Ca^2+^ influx is a conserved mechanism for axon navigation and regeneration [42]. However, while TRPC3 is known to guide growth cone turning, its direct role in promoting axon extension and the underlying molecular mechanism have remained elusive. Our study now fills this gap by demonstrating that TRPC3 drives spontaneous Ca^2+^ microdomains (Figure 1) that are both necessary and sufficient for axon outgrowth (Figures 2, 3, 5), thereby providing a mechanistic explanation for TRPC3‑dependent regenerative elongation. However, further studies are needed to determine whether the TRPC3‑dependent regenerative effects observed here also apply to other CNS injury models, such as spinal cord or optic nerve injury.

### 3.2 Exploratory Observations in the CNS With Neuroprotective Effect

Extending these findings to the central nervous system, we found that TRPC3 is highly expressed in substantia nigra dopaminergic neurons and is markedly downregulated in MPTP‐induced Parkinsonian degeneration (Figure 6A–C). Restoring TRPC3 expression—which also enhanced smCa hotspots and axon regrowth in hESC‐derived DA neurons and SH‐SY5Y cells (Figure 7G–K, Figure S14)—was associated with improved DA neuron survival, enhanced evoked dopamine release (Figure 7A–F), and ameliorated motor deficits (Figure 6F–I). These results align with emerging evidence implicating TRPC channels in neurodegeneration [43, 44], including TRPC1's reported protective effects in dopaminergic neurons [45] and TRPC6's role in synaptic resilience. Our findings add to this concept by identifying TRPC3 as a TRPC family member whose loss correlates with increased vulnerability in a PD context. Nevertheless, whether TRPC3 acts primarily by enhancing regenerative capacity, stabilizing intracellular Ca^2+^ homeostasis, or engaging neurotrophic pathways remains to be fully defined. Conditional loss‑of‑function studies and systematic pathway dissection will be essential to establish the causal role of TRPC3‑smCa signaling in vivo.

Emerging evidence supports a neuroprotective role for TRPC3, often in concert with TRPC6. For instance, TRPC3/6 promotes cerebellar granule neuron survival [44] and maintains the viability and differentiation potential of mouse embryonic stem cells [46]. Neuroprotection is also achieved via TRPC3/6/7 activation by tetrahydrohyperforin in models of Alzheimer's disease [47, 48], and via BDNF/TrkB regulation of TRPC3/6 against myocardial infarction [49]. Moreover, TRPC1/3 helps sustain intracellular Ca^2+^ homeostasis, and its loss has been linked to neurodegeneration [50]. However, detrimental effects have also been reported—for example, TRPC3 overactivation contributes to α‑synuclein‑induced olfactory dysfunction [51], highlighting that TRPC channels can exert either protective or harmful actions depending on subtype and disease context [52, 53, 54, 55]. Consequently, the current understanding of TRPC3 in neuroprotection remains incomplete, especially regarding the underlying mechanisms. Our study provides new evidence and a preliminary mechanism (Figures 6 and 7; Figure S12) that further supports the neuroprotective function of TRPC3.

Unlike global Ca^2+^ elevations that risk excitotoxicity and indiscriminate activation of downstream effectors, spontaneous near‑membrane Ca^2+^ microdomains (smCa) are spatially confined. This compartmentalization allows high local Ca^2+^ to engage specific Ca^2+^ sensors (e.g., calmodulin) and activate pro‑repair pathways without triggering bulk cytosolic overload. In the PNS, TRPC3‑driven smCa instruct axon regeneration by delivering precise Ca^2+^ signals to growth‑promoting sites (Figure S1). Extending this logic to the CNS, we propose that TRPC3‑mediated smCa confer neuroprotection by coupling pro‑survival Ca^2+^ signals to downstream effectors while avoiding harmful global transients. Thus, the precision of local Ca^2+^ signaling—rather than global Ca^2+^ amplitude—may emerge as a key determinant of both PNS regeneration and CNS neuroprotection.

### 3.3 Limitations and Future Directions

Several limitations warrant caution. First, our conclusions regarding the functional role of Ca^2+^ microdomains are based on manipulation of the TRPC3 channel, which we have previously established as a driver of these microdomains (Figure 1). Direct manipulation of the Ca^2+^ microdomains themselves will be needed to further dissect their causal contribution to neural repair. Second, while pharmacological inhibition suggests that calmodulin acts as a downstream sensor for TRPC3‐smCa (Figure 4), the precise molecular link between spontaneous Ca^2+^ microdomain activity and downstream effectors remains to be defined [56, 57]. Third, our central nervous system findings rely primarily on gain‐of‐function approaches; loss‐of‐function studies in the Parkinson's disease model and in human ESC‐derived DA neurons will be required to establish causality. Supporting observations in human ESC‐derived DA neurons are preliminary and should be interpreted as suggestive rather than mechanistic. Moreover, the use of AAV‐mediated overexpression, while demonstrating therapeutic potential, does not fully recapitulate endogenous regulation. Long‐term safety and the potential risks of channel overactivation require rigorous evaluation in future translational studies [58].

Additionally, we recognize that our study used different species across experimental systems: human ESC‑derived neurons for in vitro validation, mice for peripheral nerve injury and Parkinson's disease models, and rats for primary sensory neuron cultures. Although the core findings are consistent across these models, we cannot completely rule out species‑dependent differences in TRPC3 function or downstream signaling. Therefore, the conclusions should be interpreted with caution when extrapolated to human clinical contexts. Future studies using human tissues or patient‑derived neurons would help further validate the translational relevance.

## 4 Conclusion

Our work identifies TRPC3 as a molecular driver of Ca^2+^ microdomains that in turn function as a signaling module for peripheral nerve regeneration, with implications for neuroprotection in central dopaminergic neurons (Figure 8). By identifying TRPC3 as a molecular hub that links spontaneous, localized Ca^2+^ influx to structural and functional recovery, these findings offer a framework for understanding how spatial compartmentalization circumvents the “calcium paradox”. While clinical translation will require further validation, our results highlight the TRPC3‐smCa axis as a potential therapeutic target for traumatic nerve injury and neurodegenerative disorders.

## 5 Experimental Section

### 5.1 Animals

All animal procedures were approved by the Institutional Animal Care and Use Committee and were in accordance with the guidelines of the Association for Assessment and Accreditation of Laboratory Animal Care. Animals were maintained in a controlled environment under a 12‐h light/dark cycle at 22°C ± 2°C, with food and water provided ad libitum [59, 60]. Postnatal day 5 Sprague–Dawley rats were used for all cultured single‐cell experiments shown in Figures 1, 2, 3, 4 (except for the TRPC3 knockout experiments of C57 mice). Adult male C57 mice (8 weeks old) were used for single‐cell, tissue‐slice experiments and in vivo mechanical sensory behavioral tests in Figure 5. Adult male C57 mice (16 weeks old) were used for Parkinson's disease model‐related experiments.

### 5.2 Sensory DRG Neuron Isolation and Culture

DRG neurons were isolated and cultured as previously described [60, 61, 62]. Briefly, all DRG ganglia were collected and placed in cold L15 medium (Gibco). Attached tissue was removed, and ganglia were cut into several pieces before incubation in DMEM/F12 (Gibco) containing 0.2–0.3 mg/mL trypsin and 1 mg/mL collagenase for 40 min at 37°C under 5% CO_2_. The ganglia pieces were then washed twice with DMEM/F12 and dissociated into single cells by 8–10 gentle triturations. Cells were enriched by centrifugation (600 rpm, 5 min) and resuspended in an appropriate volume of DMEM/F12. The resulting cell suspension was plated onto 0.1% poly‐L‐lysine‐coated coverslips and maintained in Neurobasal medium supplemented with 2% B27 and 1% GlutaMAX (all from Gibco). TIRF imaging of spontaneous microdomain Ca^2+^ hotspots was performed after ∼20 h of culture. For axon regeneration experiments, drugs were added to the Neurobasal medium at the start of culture, and effects were analyzed after ∼24 h. The control group consisted of neurons cultured in standard Neurobasal medium without pharmacological agents.

### 5.3 Electrical Transfection, Plasmids, and Knockdown siRNAs

For transfection experiments, DRG neurons were collected and electroporated with TRPC3 or Lck‐GCaMP5g plasmids, or with TRPC3‐targeting siRNAs, using the Neon 100‐µL electroporation system (MPK10096, Invitrogen). To ensure consistent plasmid expression, cell density, plasmid concentration, electroporation parameters, and culture duration were carefully controlled across experiments. TRPC‐targeting and scramble siRNAs (Table 1) were purchased from Beijing Tsingke Biotech Co., Ltd.

**TABLE 1:** TRPC siRNAs.

siRNA	sense (5’→3’)	antisense (5’→3’)
TRPC1	CCAGGUUUCGUCUUGAUAU	AUAUCAAGACGAAACCUGG
TRPC3‐1	GCUGCGCAUUGCCAUAAAU	AUUUAUGGCAAUGCGCAGC
TRPC3‐2	GGUUCAGUAUUUCACCUAU	AUAGGUGAAAUACUGAACC
TRPC6	GCGAUGAUCAACAGUUCAU	AUGAACUGUUGAUCAUCGC

### 5.4 High‐Resolution TIRF Imaging in Live Cells

TIRF live imaging was performed as previously described [36, 60]. Imaging was carried out on an inverted microscope equipped with a 100× TIRF objective lens (Olympus IX‐81, NA 1.45), and images were captured using an Andor EMCCD camera with ∼50 ms exposure via Andor iQ software. Spontaneous Ca^2+^ activity or Synaptophysin‐pHluorin‐labeled quantal vesicle release was recorded for 60 s.

For Ca^2+^ imaging, regions of spontaneous microdomain Ca^2+^ activity were identified based on the spatial and temporal coupling of individual hotspots [12]. Connected local Ca^2+^ transients occurring within the same time window were counted as a single region of interest (ROI; see Movies S1 and S2). To minimize potential contributions from TRPA1‐expressing neurons—which are predominantly restricted to small‐diameter sensory subsets—we selectively targeted medium‐ and large‐diameter DRG neurons for recording [13, 63]. For Synaptophysin‐pHluorin recordings, exocytotic events were distinguished from vesicle movement by defining events as abrupt fluorescence increases at pHluorin puncta, immediately followed by a return to baseline or lower fluorescence levels.

All recordings were performed at room temperature (∼25°C). The recording solution contained (in mmol/L): 2 CaCl_2_, 1 MgCl_2_, 150 NaCl, 5 KCl, 10 H‐HEPES, and 10 D‐glucose, pH 7.4. Fluorescence TIRF images were analyzed using ImageJ and processed in Adobe Photoshop.

### 5.5 Acute Slice Preparation

Acute brain slices were prepared following established procedures [12, 64] with minor adaptations. Mice were anesthetized with avertin (10 mg/kg, i.p.) and transcardially perfused with ∼5 mL of ice‐cold cutting artificial cerebrospinal fluid (aCSF). The cutting aCSF contained (in mmol/L): 110 C_5_H_14_NClO, 2.5 KCl, 0.5 CaCl_2_, 7 MgCl_2_, 1.3 NaH_2_PO_4_, 25 NaHCO_3_ and 25 glucose, and was continuously bubbled with 95% O_2_ / 5% CO_2_. Following perfusion, brains were rapidly removed and sectioned into 300‐µm coronal slices using a vibratome (Leica VT1200s). Slices containing the substantia nigra pars compacta (SNc) and striatum were collected and incubated in recording aCSF at 37°C for 30 min, followed by an additional 30 min at room temperature. Recording aCSF consisted of (in mmol/L): 125 NaCl, 2.5 KCl, 2 CaCl_2_, 1.3 MgCl_2_, 1.3 NaH_2_PO_4_, 25 NaHCO_3_ and 10 glucose.

Slices were transferred to a recording chamber and continuously superfused with recording aCSF at 2 mL/min. Neurons were visualized using an Olympus BXWI51 microscope equipped with infrared differential interference contrast optics and an IR‐1000 camera.

### 5.6 CFE Amperometric Recording of Dopamine Release in Brain Slices

Amperometric measurements of dopamine (DA) release in striatal slices were performed using carbon‐fibre electrodes (CFEs) following established protocols [64, 65]. CFEs (7‐µm diameter, ∼200‐µm exposed tip) were positioned with the sensing surface fully inserted beneath the slice surface at an angle of ∼30°. A constant potential of +780 mV was applied using an EPC10/2 amplifier controlled by PatchMaster software (HEKA Elektronik). Electrical stimulation was delivered through a bipolar platinum electrode (150‐µm diameter) connected to a Grass S88K stimulator (Astro‐Med). Either single pulses (0.2 ms, 0.6 mA) or burst paradigms (20 pulses at 10 Hz) were used to evoke DA release. Amperometric currents were low‐pass filtered at 100 Hz and digitized at 3.13 kHz. Data were analyzed offline in Igor Pro (WaveMetrics) to quantify event amplitude and charge transfer.

### 5.7 Surgery of Sciatic Nerve Cut‐off Model

The sciatic nerve cut‐off model was adapted from a conventional sciatic nerve injury model [64]. Briefly, mice were anesthetized with Avertin, and hair on the left thigh was carefully shaved. The sciatic nerve was exposed through a small skin incision, and approximately 3 mm of the tibial and common peroneal nerves were transected, while the sural nerve was preserved. Importantly, the nerves were not ligated with nylon sutures. For the sham procedure, the surgical steps were identical except that the nerves were left intact. The incision was then closed with nylon sutures, and the wound was disinfected with iodophor. Postoperatively, mice were maintained under a 12‐h light/dark cycle at 22°C ± 2°C, with food and water provided ad libitum. Mice were subsequently anesthetized with Avertin for sciatic nerve collection and analysis.

### 5.8 Antibodies

Primary antibodies: Mouse anti‐Tuj1 1:500 (Sigma, T8578), Rabbit anti‐SCG‐10 1:200 (Immunoway, YT5076), Rabbit anti‐TRPC3 1:200 (Immunoway, YT5520). Mouse anti‐TH antibody 1:1000 (SYSY, 213111). Mouse anti‐NeuN 1:200 (Proteintech, 66836‐1‐Ig). Secondary antibodies: Alexa Fluor 594, Donkey anti‐mouse IgG (H+L) 1:1000 (Invitrogen, A‐21203); Alexa Fluor 488, Donkey anti‐rabbit IgG (H+L) 1:1000 (Invitrogen, A‐21206); Alexa Fluor 488, Donkey anti‐mouse IgG (H+L) 1:1000 (Invitrogen, A‐21202); Alexa Fluor 594, Donkey anti‐ rabbit IgG (H+L) 1: 1000 (Invitrogen, A‐21207); Alexa Fluor 647, Donkey anti‐ rabbit IgG (H+L) 1: 1000 (Invitrogen, A‐32795). DAPI (Biosharp, BL105A).

### 5.9 Immunofluorescence of Single Cells and Image Processing

For immunostaining of cultured DRG neurons [66], cells were washed three times with PBS and fixed in 4% paraformaldehyde for 30 min. Cells were then permeabilized with 0.3% Triton X‐100 in PBS for 5 min at room temperature. After blocking with 2% BSA in PBS for 1 h, cells were incubated with primary antibodies for 1 h at room temperature or overnight at 4°C. Following three washes with blocking solution, cells were incubated with secondary antibodies for 1 h. Cells were then washed three times with blocking solution and once with PBS before mounting on slides with 50% glycerol. Fluorescence images were acquired using a Leica STELLARIS 5 microscope with Leica LAS X software (Leica) and analyzed offline using ImageJ and Adobe Photoshop. All immunofluorescence images were acquired and processed under identical settings. For presentation, brightness and contrast were linearly adjusted, and minor background debris not overlapping with cells was manually excluded to improve clarity. These operations were applied consistently across all experimental groups and did not influence the quantitative analysis (Figure S6D).

### 5.10 Immunofluorescence of Tissue Slices

For tissue slice immunostaining, tissues were collected and fixed in 4% paraformaldehyde at 4°C for 24 h. Following sequential dehydration in 10%, 20%, and 30% sucrose, 20‐µm slices were cut along the sciatic nerve using the RWD FS900. Slices were washed three times with PBS for 10 min each and permeabilized with 0.3% Triton X‐100 in PBS for 15 min at room temperature. After blocking with 10% NDS in PBS for 2 h, slices were incubated with primary antibodies overnight at 4°C. Slices were then washed three times with blocking solution and incubated with secondary antibodies for 2 h. After two additional washes, slices were mounted on slides with DAPI/Antifade solution (S7113, Sigma). Fluorescence images were acquired using a Leica STELLARIS 5 microscope with Leica LAS X software and analyzed offline using ImageJ and Adobe Photoshop.

### 5.11 Measurement of Regenerated Axons’ Length

For cultured single DRG neurons, soma and axons were labeled with the Tuj1 antibody or EGFP. The longest axon branch of each cell was measured for quantification using ImageJ (Figure S6D). DRG neurons lacking axons were also included to determine the percentage of axon regeneration. For in vivo sciatic nerve regeneration, existing axons were labeled with Tuj1, and newly regenerated axons were labeled with SCG10 [22]. The length of SCG10‐positive fibers was measured for quantification using ImageJ.

### 5.12 Viral Delivery and Induction of the MPTP Parkinson's Disease Model

Adult male C57BL/6J mice (16 weeks old) were used for all experiments. A Parkinson's disease (PD) model was induced by intraperitoneal administration of MPTP (30 mg/kg/day) for five consecutive days. On day 6, mice were anaesthetized with Avertin and secured in a stereotaxic frame. Adeno‐associated virus (AAV) carrying TRPC3 or a control construct under the dopaminergic neuron–specific tyrosine hydroxylase (TH) promoter was bilaterally delivered into the substantia nigra pars compacta (SNc) at the following coordinates relative to bregma: anteroposterior −3.0 mm, mediolateral ±1.2 mm and dorsoventral −4.5 mm. A total volume of 150 nL per site (4 × 10^12^ genome copies/mL) was injected at 0.1 µL/min, and the needle was left in place for 5 min to permit diffusion. Mice recovered and expressed the viral constructs for 3 weeks before behavioral assays, histological analysis and electrophysiological recordings.

### 5.13 Differentiation of Human ESC‐Derived Neural Progenitor Cells (NPCs) Into Dopaminergic Neurons

Human ESC‐derived neural progenitor cell lines (NPCs) were thawed and expanded on Laminin‐521‐coated plates in Neurobasal medium supplemented with 1% N‐2, 2% B27, 1 µm SAG, and 2 µm CHIR99021 [27]. NPCs were then patterned toward a midbrain dopaminergic fate by culturing them for 5–7 days in Neurobasal medium containing 2% B27, 0.1 µm RO4929097, 10 ng/mL BDNF, 10 ng/mL GDNF, and 1 µm DMH1. Following midbrain patterning, cells were further differentiated into dopaminergic (DA) neurons in Neurobasal medium supplemented with 2% B27, 5 µm forskolin, 10 ng/mL BDNF, 10 ng/mL GDNF, and 1 µm DMH1 for an additional 4–5 weeks, with medium changes every 2–3 days. Morphological maturation was confirmed by the emergence of long, highly branched axon characteristic and TH expression of mature DA neurons (Figure S13).

Fully differentiated DA neurons were then dissociated into single cells and electroporated with TRPC3, Lck‐GCaMP5g, or EGFP plasmids for spontaneous microdomain Ca^2+^ hotspot imaging and axon regeneration assays after 24 h of expression.

### 5.14 Behavioral Tests

Behavioral assessments were performed in accordance with procedures outlined in prior reports [12, 64, 67, 68, 69, 70, 71]. To facilitate environmental habituation, mice were transferred from their home cages to the testing room 1 h before the onset of behavioral testing. Unless otherwise noted, animal behavior was recorded using an overhead camera coupled with the Smart video tracking software (Panlab Inc.). The testing environment was maintained under dim illumination (∼20 lux) to reduce anxiety‐related responses.

### 5.15 Locomotion Test

Each mouse was placed in the central zone of an open‐field arena (40 × 40 × 40 cm) and permitted to freely explore the environment. Locomotor behavior was monitored for 30 min using an overhead camera in conjunction with the Smart video tracking software (Panlab Inc.). Total distance traveled during the session was quantified as an index of locomotor activity. Following each trial, the apparatus was thoroughly cleaned with 75% ethanol to eliminate residual odors.

### 5.16 Rota‐Rod Test

Mice were positioned on the rotating rod (Med Associates) with the device operating in an accelerating mode (4–40 rpm). The latency to fall during each trial was automatically detected and recorded by the system. Animals underwent three trials, with an inter‐trial interval of at least 15 min. Raw performance data were collected and subsequently processed in Microsoft Excel. For each animal, fall latency was calculated as the mean value across the three trials.

### 5.17 Grip Strength Test

Grip strength was assessed using a grip strength meter (Ugo Basile, 47200). Briefly, each mouse was held by the tail and lowered until its four limbs firmly grasped the T‐bar attached to the digital force transducer. The animal was then gently lowered to a horizontal position and gradually pulled backward until it released its grip, at which point the peak force (in grams) was recorded. Each mouse completed five trials with a 5‐min interval between measurements.

### 5.18 Cylinder Test

Briefly, mice were placed in a transparent Plexiglas cylinder (12 cm in diameter, 30 cm in height) positioned in front of two mirrors, and their behavior was video‐recorded for 10 min. Recorded videos were analyzed offline by an observer blinded to the experimental conditions. Each rearing event was evaluated for both the number and duration of contacts made with the inner wall of the cylinder using the forepaws.

### 5.19 Mechanical Sensitivity Behavior Test

Von Frey testing was performed as previously described [12, 64] using Dixon's up–down method. The paw withdrawal threshold (PWT, g) was used to assess mechanical pain sensitivity. Prior to testing, mice were placed in a transparent compartment (7 × 9 × 7 cm) on an elevated metal mesh for ∼15 min to acclimate. A series of von Frey filaments (0.008–2.0 g) were applied perpendicularly to the central anterior region of the hind paw plantar surface until the filament bent into a “C” shape (∼2 s). Rapid paw withdrawal, licking, flinching, or shaking were considered positive responses. Testing began with the 0.008 g filament. If no response occurred, the next higher filament was applied; if a positive response occurred, the previous lower filament was used. Five positive responses were recorded, and the PWT was calculated as the filament producing a 50% likelihood of inducing paw withdrawal.

### 5.20 Statistical Analysis

All experiments included appropriate side‐by‐side controls and were performed in random order [60, 72]. Each experiment was replicated at least three times. Sample sizes were consistent with those reported in comparable studies, and all successfully measured samples or recordings were included in the analysis. Data are presented as mean ± S.E.M., with all data points shown. Normality was assessed prior to statistical analysis. For normally distributed data, two‐tailed Student's *t*‐tests were used for comparisons between two unpaired groups, and paired Student's *t*‐tests were used for paired groups. For non‐normally distributed data, the Mann–Whitney test was applied for two unpaired groups, and the Wilcoxon matched‐pairs signed‐rank test was used for paired groups. Comparisons involving more than two groups were performed using One‐way ANOVA with appropriate post‐hoc multiple‐comparison tests. All statistical analyses were conducted using GraphPad Prism 9 (GraphPad Software, Inc.), and differences were considered significant at *p* < 0.05.

## Author Contributions

R.H., Y.L., J.Z., Z.X., Y.W., and R.W. performed and analyzed the experiments with the help of B.W., C.S., Y.C., J.Y., Z.L., H.X., and Z.C.; R.H., C.W., and Z.Z. designed the work and wrote the manuscript; R.H. supervised the project.

## Funding

This work was supported by the National Natural Science Foundation of China (32400650, 82201404, 32525031, 32171233, 32571210), the Clinical Research Special Project of Shanghai Municipal Health Commission (20244Y0148), the Sanqin Talent Special Support Program (2024STD04), the Fund of Shaanxi Province of China (2023SYJ09), the Natural Science Foundation of Shaanxi Province of China (2024JC‐YBMS‐141, 2024JC‐YBMS‐146, 2026JC‐YXQN‐067).

## Ethic Statement

All experimental protocols were reviewed and approved by the Institutional ethics committee (approval no. XJTU Life Sciences (2025)‐35, 2026‐DWSB‐088). No human embryos or subjects were used in this study.

## Conflicts of Interest

The authors declare no conflicts of interest.

## Supporting information




**Supporting File 1**: advs77156‐sup‐0001‐SuppMat.pdf.

**Supporting File 2**: advs77156‐sup‐0002‐SuppMat.pdf.

**Supporting File 3**: advs77156‐sup‐0003‐MovieS1.mp4.

**Supporting File 4**: advs77156‐sup‐0004‐MovieS2.mp4.

## Data Availability

The data that support the findings of this study are available from the corresponding author upon reasonable request.
