# Supplementary material for: TRPC3‐Driven Calcium Microdomains Instruct Peripheral Nerve Regeneration With Protective Implications for Central Neurons
Source: Adv Sci (Weinh). 2026 Aug 25;13(53):e77156. doi: 10.1002/advs.77156 (PMC13505323; doi:10.1002/advs.77156)
Supplement: Supplementary file 1 [file ADVS-13-e77156-s002.pdf]

TRPC3-driven Calcium Microdomains Instruct Peripheral Nerve Regeneration with Protective Implications for Central Neurons

Supplementary Figures and Legends:

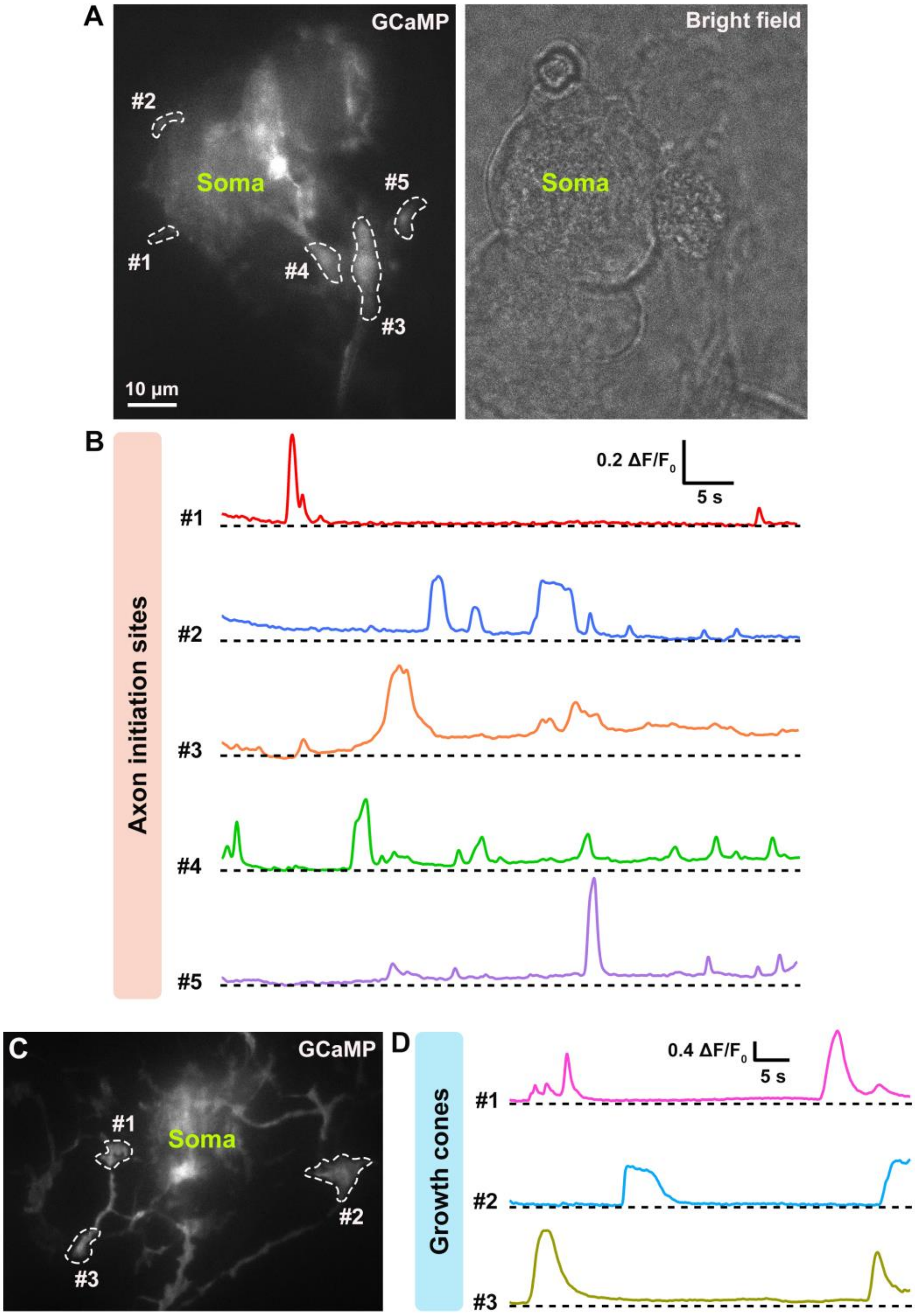

FIGURE S1. smCa activities exist in axon initiation sites and growth cones.

(A) Left: TIRF image of a rat DRG neuron cultured for 20 h and transfected with

Lck-GCaMP5g. Boxed regions indicate axon initiation sites. Right: Bright-field image of the same cell.

(B) Time-course traces of fluorescence intensity from the five boxed regions shown in (A).

(C, D) Same analyses as in (A, B), but with boxed regions focused on growth cones.

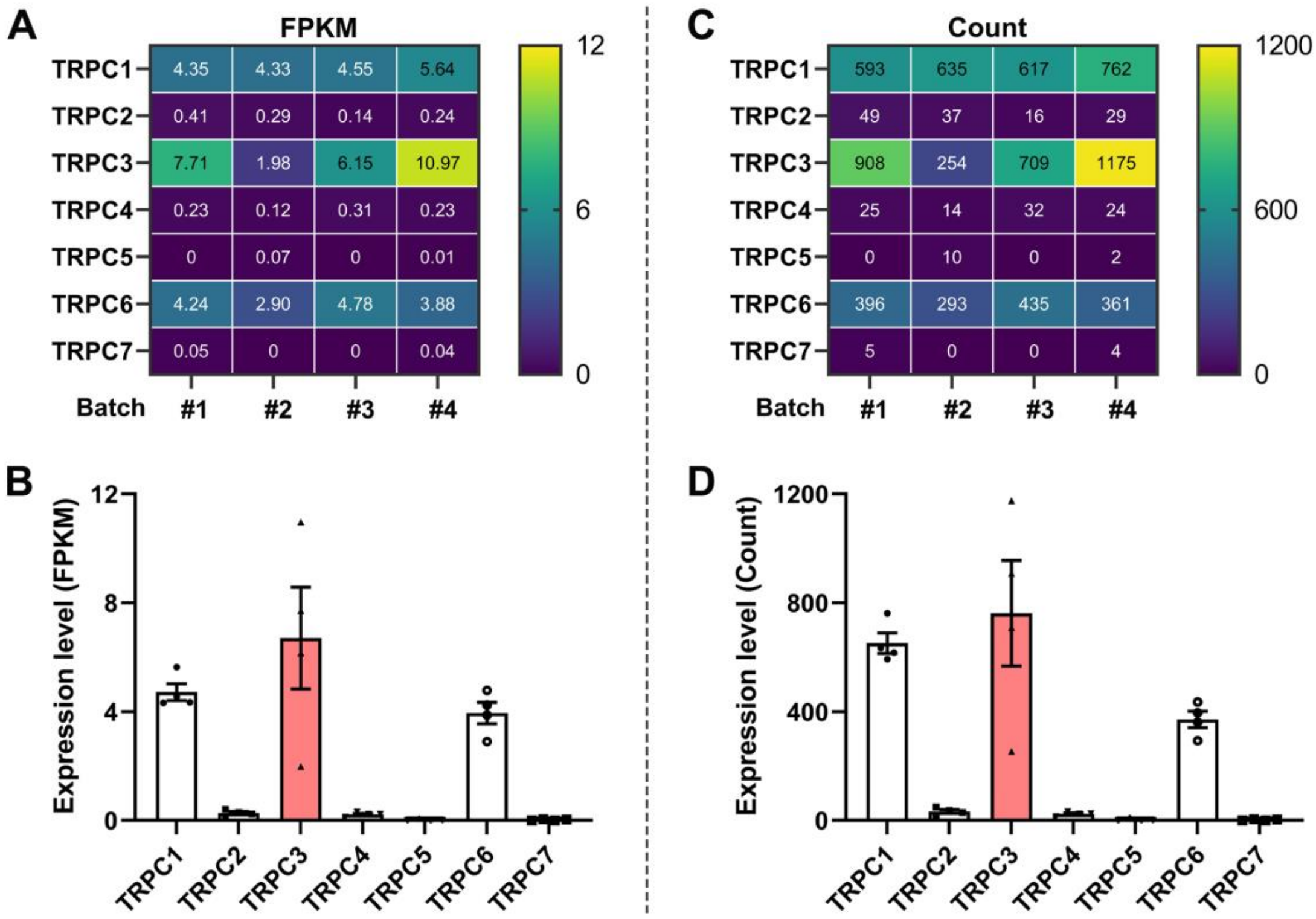

FIGURE S2. Bulk RNA-seq analysis of TRPC1–7 channel expression in rat DRG neurons.

(A) Heat map showing RNA expression levels (FPKM) of the seven TRPC channels in 20-h cultured rat DRG neurons.

(B) Quantification of FPKM values for TRPC1–7 shown in (A).

(C, D) Same analyses as in (A, B), but presenting RNA expression levels as read counts. Data are shown either as heat maps with all values indicated or as bar charts presenting mean ± s.e.m., with all data points included. n = 4 replicates.

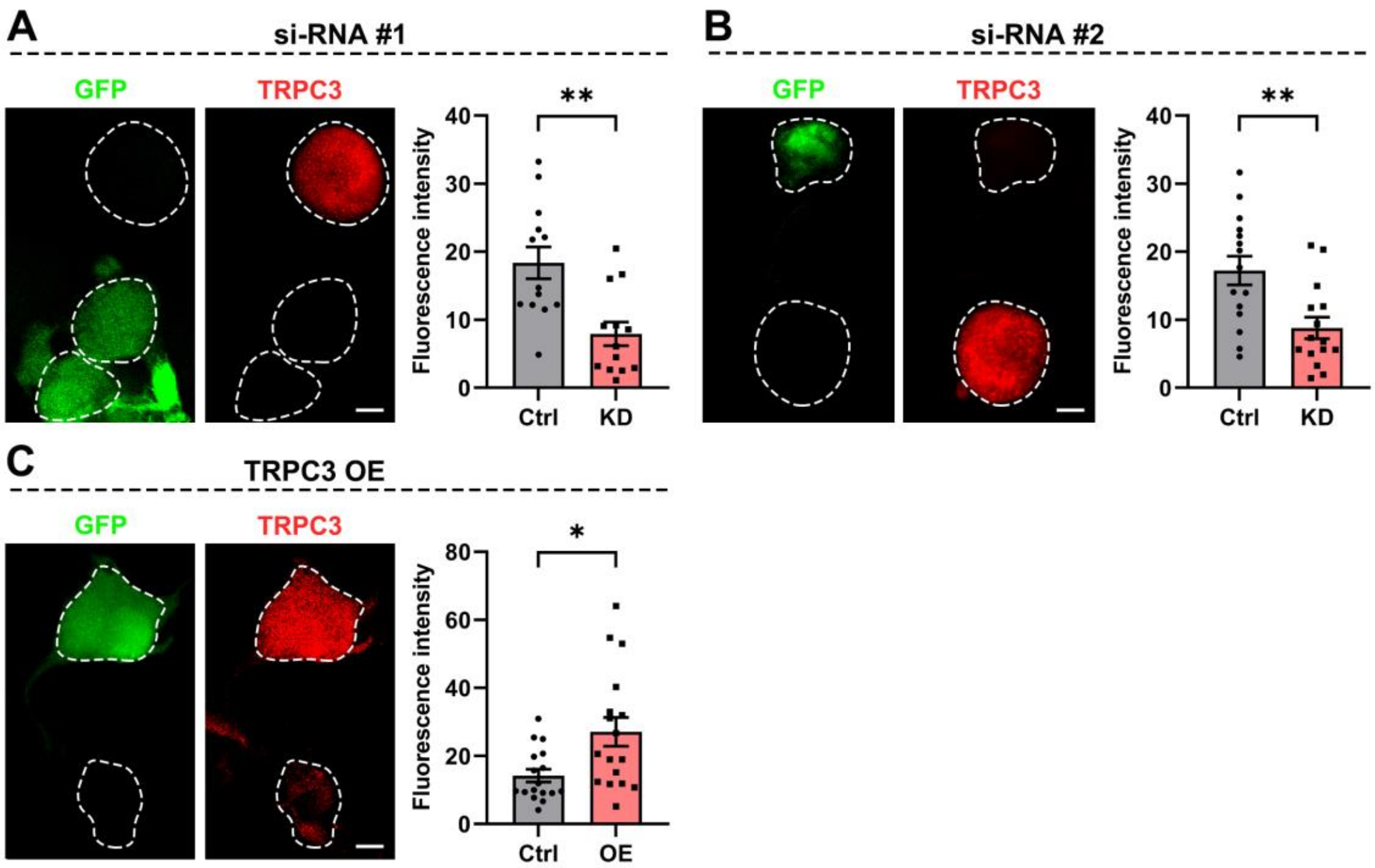

FIGURE S3. The TRPC3 KD and OE efficiency validation.

(A, B) Left: Immunofluorescence images of rat DRG neurons in control and TRPC3 KD (two independent siRNAs) groups. Right: Quantification of membrane TRPC3 expression intensity. Scale bar, 10 µm. n = 13 cells per group in (A), 15 cells per group in (B).

(C) Left: Immunofluorescence images of rat DRG neurons in control and TRPC3 OE groups. Right: Quantification of membrane TRPC3 expression intensity. Scale bar, 10 µm. n = 17 cells per group.

Data are presented as mean ± s.e.m., with all data points shown. Each data point represents one neuron. Statistical tests: Student's *t* test. *P < 0.05; **P < 0.01.

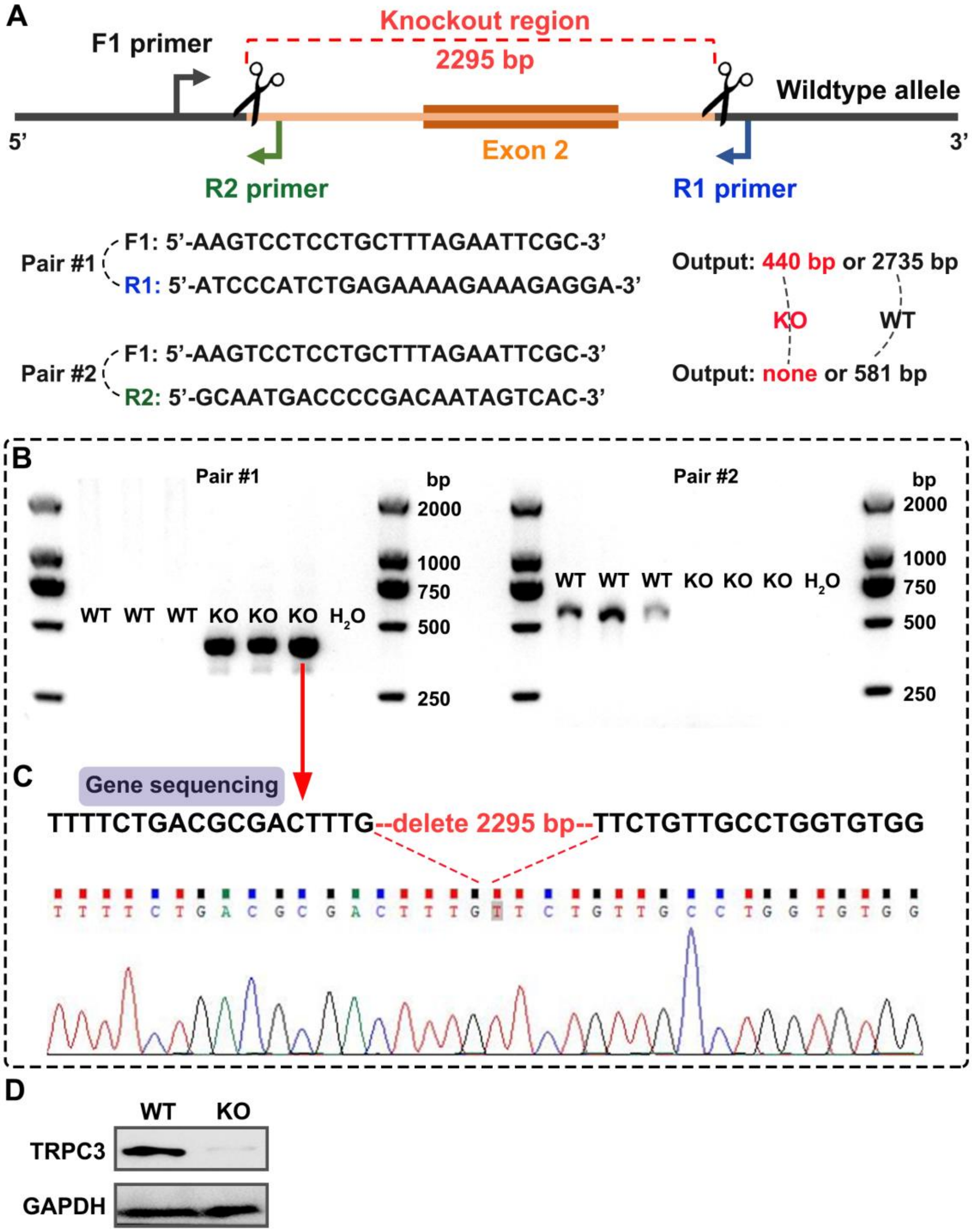

FIGURE S4. Generation and validation of TRPC3 knockout mice.

(A) Schematic of the TRPC3 targeting strategy (top), along with the primer sequences used for genotyping and the expected PCR products (bottom).

(B) Representative genotyping PCR gel showing the identification of wild-type and homozygous knockout alleles.

(C) DNA sequencing confirmation of the TRPC3 genomic locus in wild-type and knockout mice.

(D) Western blot analysis of TRPC3 protein expression in DRG tissues from wild-type and TRPC3 knockout mice, confirming loss of TRPC3 protein in the knockout samples.

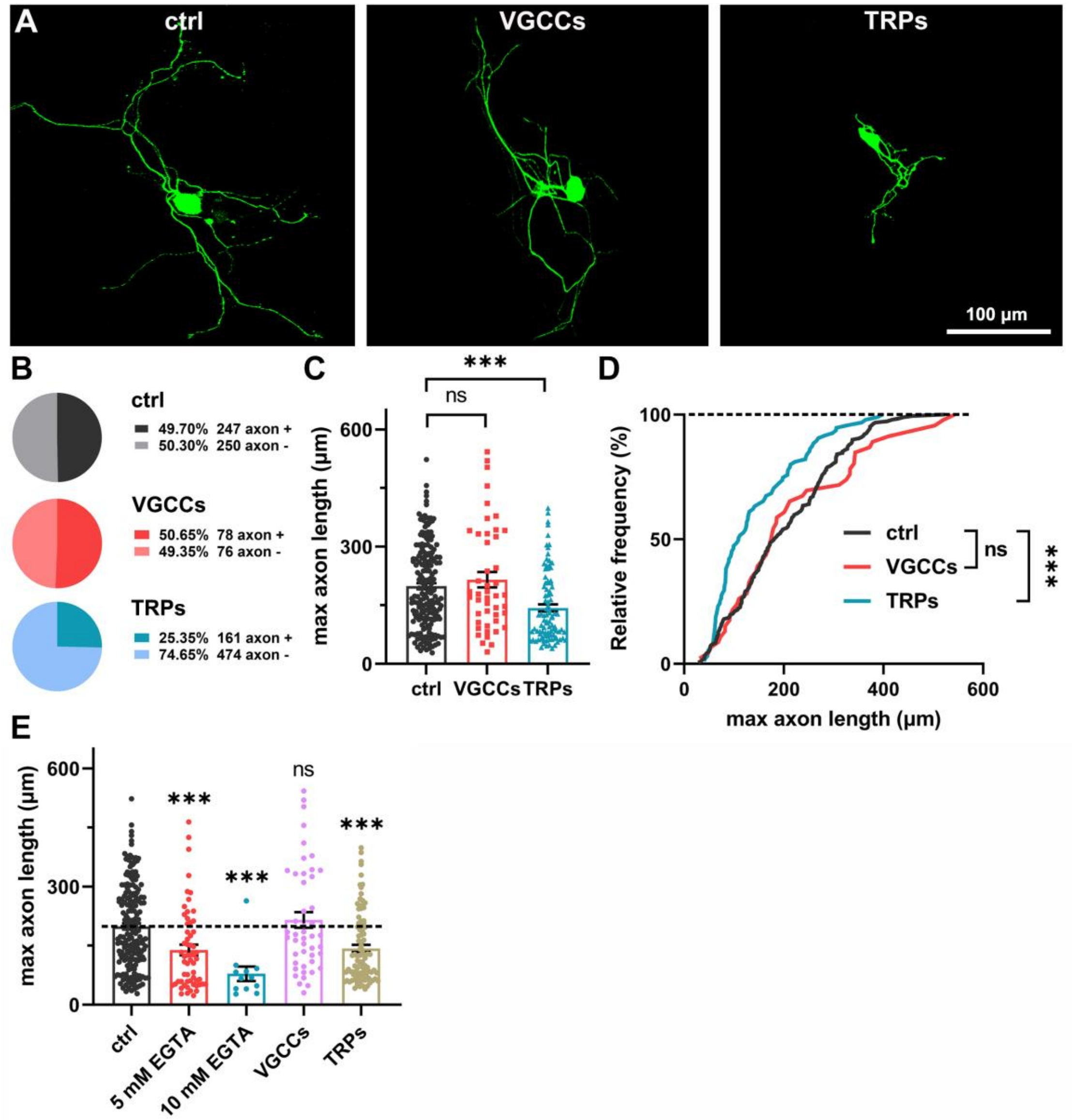

FIGURE S5. Sensory nerve regeneration depends on TRP channels.

(A) Confocal images of 24-h cultured rat sensory DRG neurons grown in control medium, medium supplemented with voltage-gated Ca^{2+} channel blockers (VGCCs: 1 μM GVIA for N-type, 5 μM nifedipine for L-type, and 1 μM TTA-A2 for T-type channels), or medium supplemented with TRP channel blockers (TRPs: 50 μM ruthenium red, a non-selective TRP antagonist).

(B) Percentage of regenerated-axon-positive cells in the control, VGCCs, and TRPs groups shown in (A).

(C) Quantification of maximum regenerated-axon length per cell in the groups shown in (A).

(D) Relative frequency distribution of maximum regenerated-axon length per cell in the control, VGCCs, and TRPs groups.

(E) Summary of maximum regenerated-axon length per cell in different drug treatment groups, which are performed in same batches.

Data are presented as mean ± s.e.m., with all data points shown. Each data point represents one neuron, and the n value is showed in the percentage panel. One-way ANOVA was used for statistical analysis. ns, not significant; ***P < 0.001.

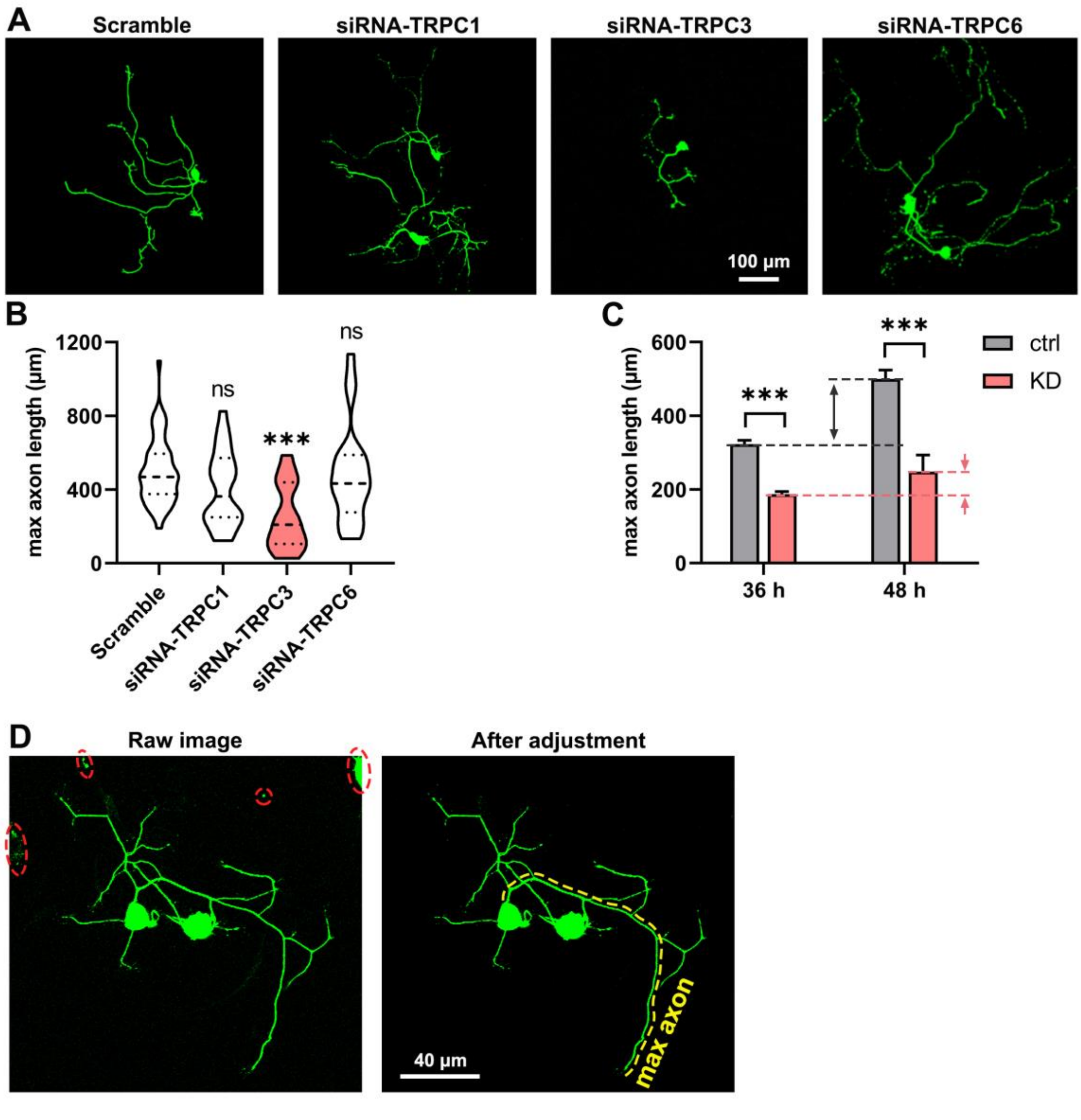

FIGURE S6. TRPC3 knockdown dramatically reduces axon regeneration.

(A) Confocal images of 48-h cultured rat sensory DRG neurons in control (scramble

siRNA) and TRPC1-, TRPC3-, or TRPC6-knockdown (TRPC-siRNA) groups.

(B) Quantification of maximum regenerated-axon length per cell in the groups shown in (A).

(C) Comparation of maximum regenerated-axon length per cell in 36 h and 48 h cultured groups.

(D) Illustration of the image-processing workflow and measurement of maximum axon length.

Data are displayed as violin plots with median and quartiles or mean ± s.e.m. Statistical analysis was performed using one-way ANOVA (B) or two-way ANOVA (C). ns, not significant; ***$P < 0.001$.

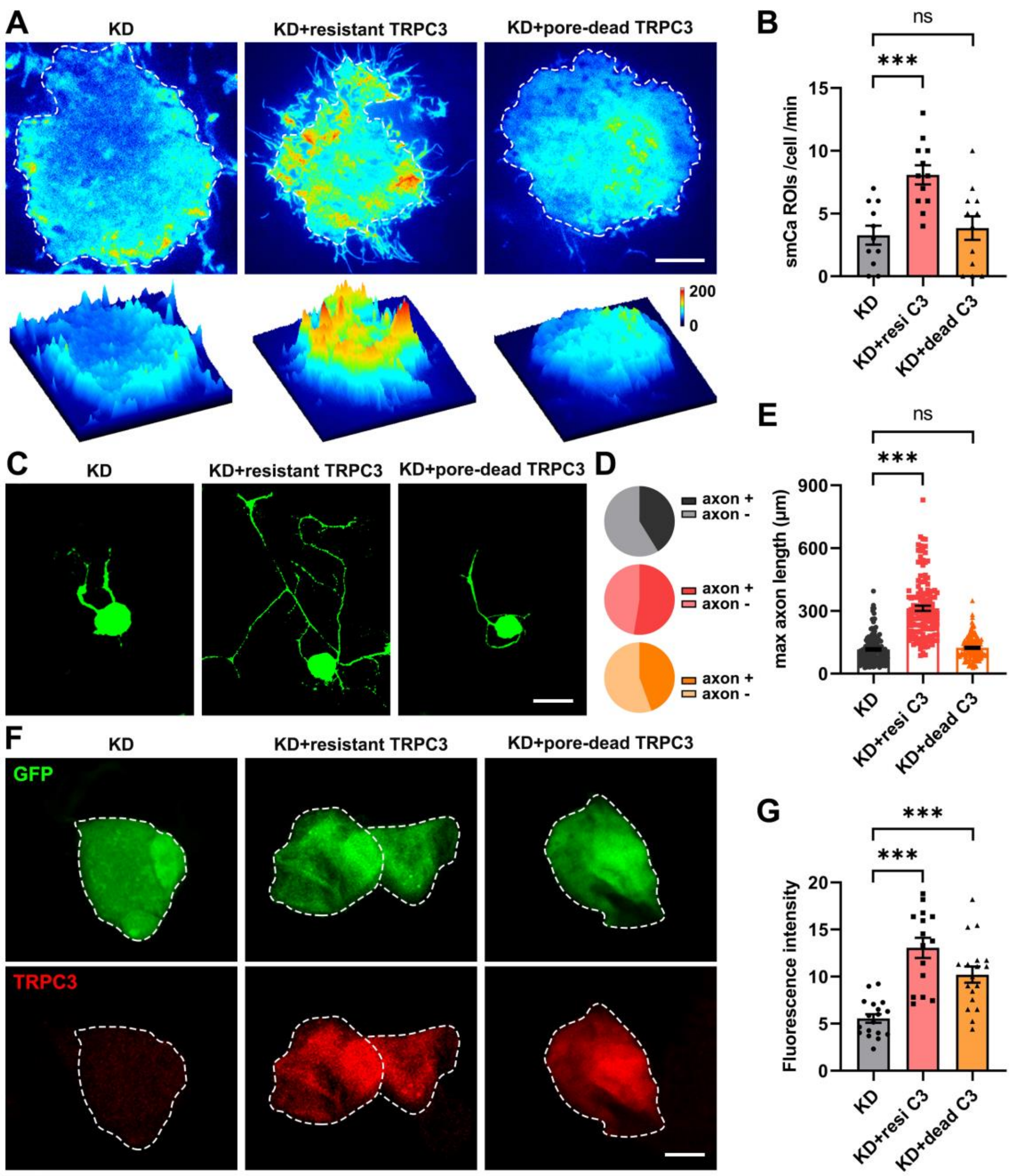

FIGURE S7. Effects of siRNA-resistant TRPC3 and pore-dead TRPC3 overexpression on smCa and axon regeneration.

(A) Top: TIRF images of rat DRG neurons cultured for 20 h and transfected with Lck-GCaMP5g in TRPC3 KD, KD + siRNA-resistant TRPC3 (hTRPC3) OE, and KD + pore-dead (E630Q) TRPC3 OE groups. Scale bar, 10 µm. Bottom: Corresponding surface plots for each group.

(B) Quantification of smCa ROI number per cell per minute. n = 11, 12, 12 cells per group, respectively.

(C) Representative images of rat DRG neurons cultured for 36 h in the same groups as

described above. Scale bar, 50 µm.

(D) Quantification of the proportion of regenerated-axon positive neurons.

(E) Quantification of maximum regenerated-axon length per neuron. n = 147, 139, 136 cells per group, respectively.

(F, G) Immunofluorescence images (F) and quantification (G) of TRPC3 expression efficiency in the indicated groups. Scale bar, 20 µm. n = 18, 15, 18 cells per group, respectively.

Data are presented as mean ± s.e.m., with all data points shown. Each data point represents one neuron. One-way ANOVA was used for statistical analysis. ns, not significant; ***P < 0.001.

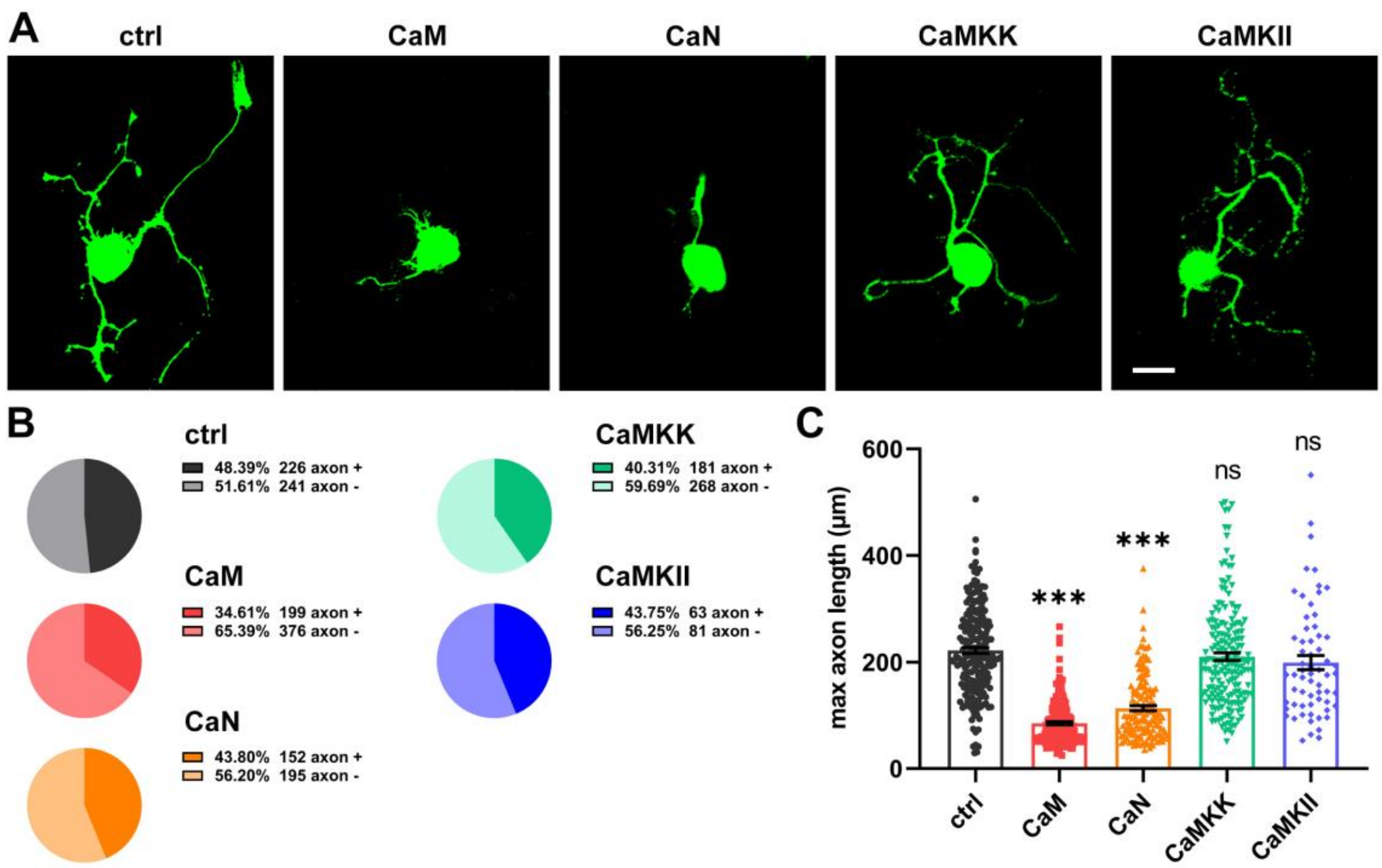

FIGURE S8. Effects of calmodulin-related signaling pathways on rat DRG axon regeneration.

(A) Immunofluorescence images of rat DRG neurons cultured for 24 h under different treatment conditions: control, CaM inhibitor (0.8 µM R24571), calcineurin (CaN) inhibitor (5 µM FK506), CaMKK inhibitor (4 µM STO609), and CaMKII inhibitor (5 µM KN93). Scale bar, 50 µm.

(B) Quantification of the proportion of regenerated-axon positive neurons in each

treatment group.

(C) Quantification of maximum regenerated-axon length per neuron in each treatment group.

Data are presented as mean ± s.e.m., with all data points shown. Each data point represents one neuron, and the n value is showed in (B) panel. One-way ANOVA was used for statistical analysis. ns, not significant; ***P < 0.001.

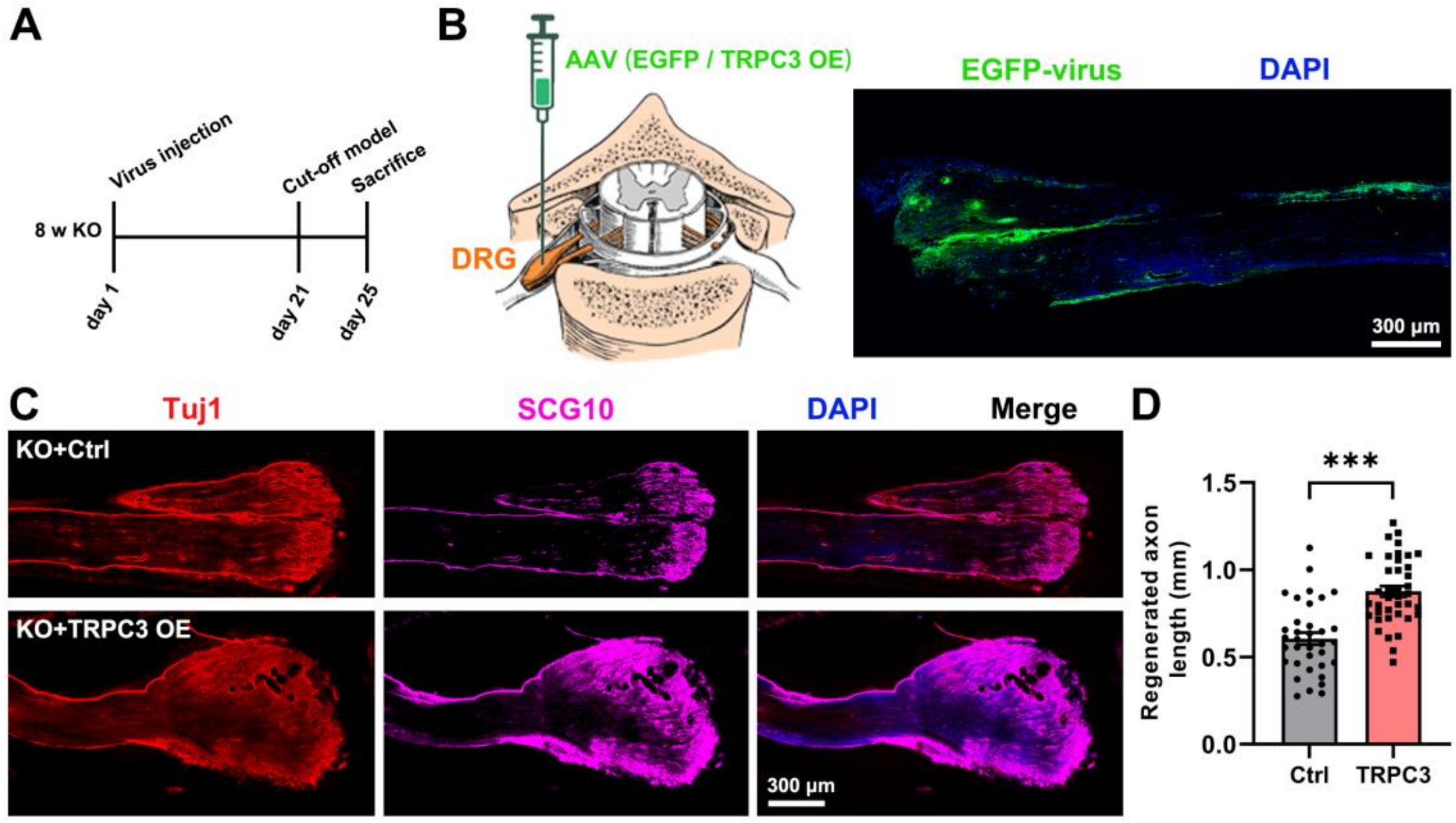

FIGURE S9. TRPC3 overexpression in DRG tissues of TRPC3 knockout mice enhances axon regeneration after sciatic nerve injury.

(A) Schematic diagram of the experimental timeline.

(B) Left: Schematic of AAV injection into DRG (L4–L6). Right: Immunofluorescence image of a sciatic nerve slice showing virus infection and expression. The image of spinal cord structure was modified from D. Michael McKeough's work.

(C) Immunofluorescence images of sciatic nerve slices from TRPC3 KO mice injected with control virus or TRPC3 OE virus, collected 4 days after sciatic nerve cut-off surgery.

(D) Quantification of regenerated axons length. n = 35, 39 fibers per group, respectively. Data are presented as mean ± s.e.m., with all data points shown. Each data point represents one regenerated fiber. Student's *t* test. ***P < 0.001.

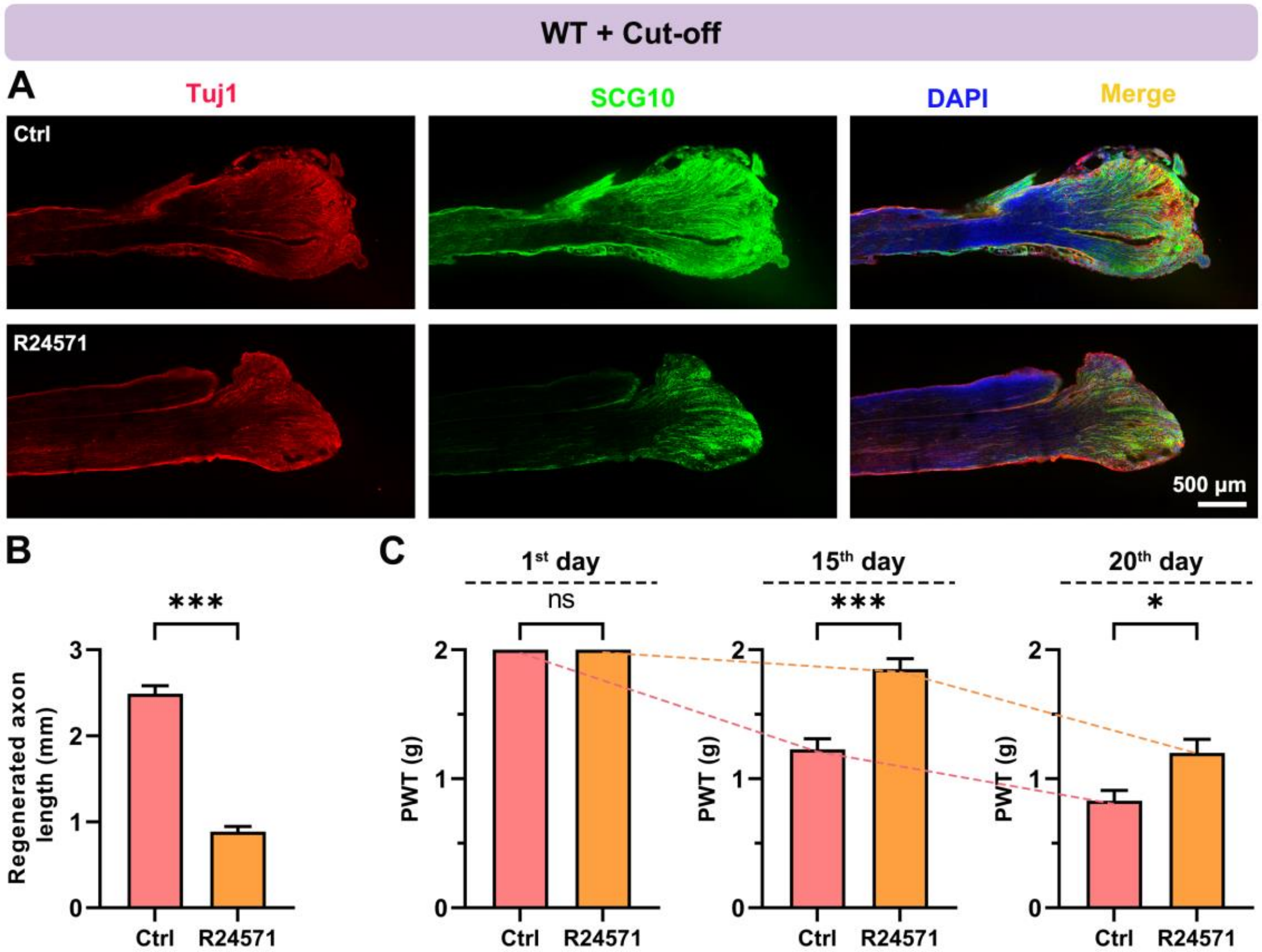

FIGURE S10. Effect of CaM blockade on axon regeneration and mechanical sensory recovery in vivo.

(A) Immunofluorescence images of sciatic nerve slices from control and CaM inhibitor-treated mice, collected 4 days post-surgery.

(B) Quantification of the regenerated axon length. n = 26 for ctrl, 31 for R24571.

(C) Time course of mechanical sensory threshold recovery in mice measured at 1st, 15th, and 20th days post-surgery, with daily intramuscular injection of CaM inhibitor at the surgical site. n =7 mice for ctrl, 8 mice for R24571.

Data are presented as mean ± s.e.m. Student's *t* test. ns, not significant; *P < 0.05; ***P < 0.001.

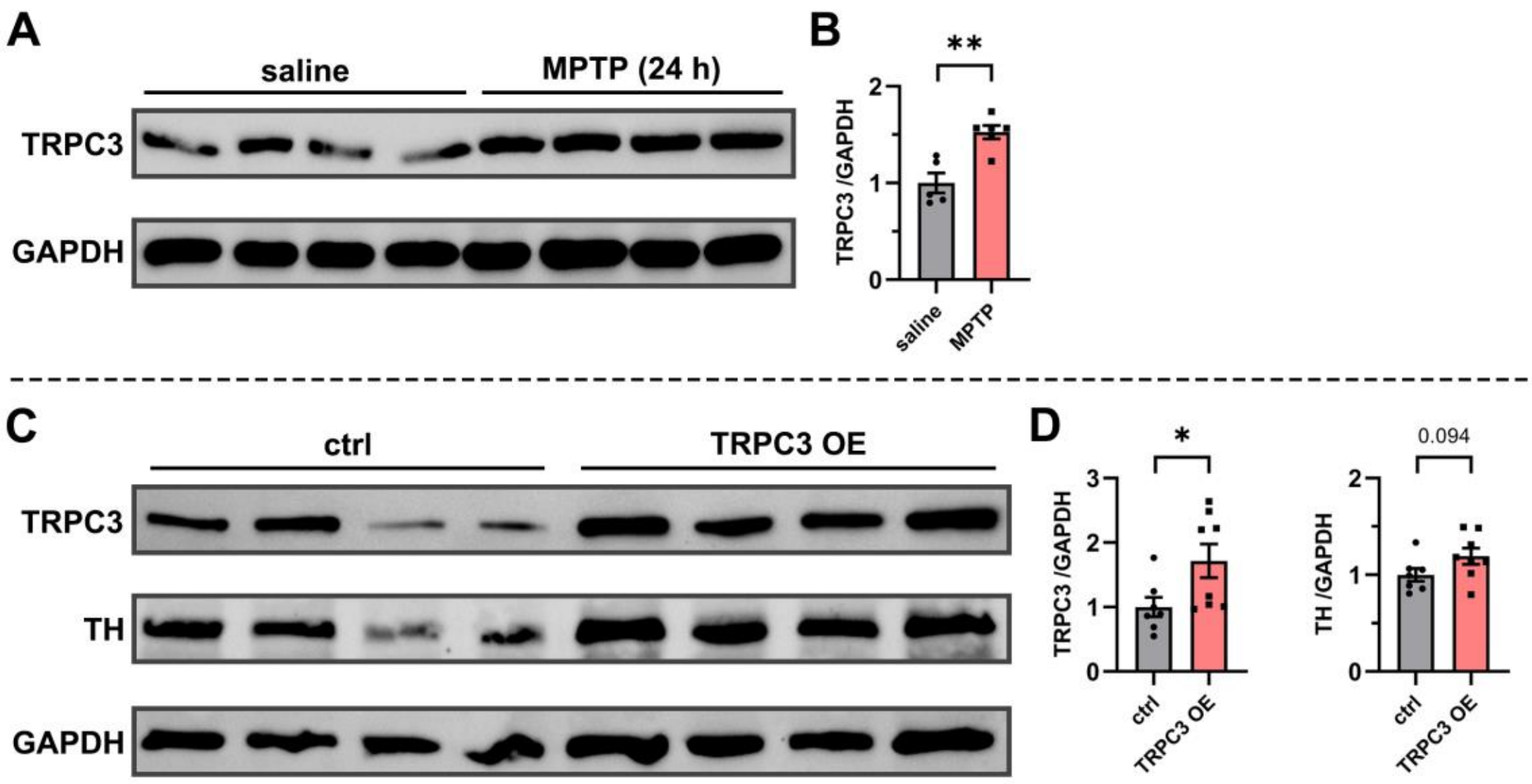

FIGURE S11. Acute MPTP treatment and TRPC3 overexpression increase TRPC3 protein levels in the substantia nigra pars compacta (SNc).

(A) Western blot analysis of TRPC3 in SNc tissue lysates from mice treated with MPTP, collected 24 h post-injection. GAPDH serves as the loading control.

(B) Quantification of TRPC3 protein levels, normalized to GAPDH.

(C) Western blot analysis of TRPC3 and tyrosine hydroxylase (TH) in SNc tissue lysates from mice injected with control or TRPC3-overexpressing virus, collected 21 days post-injection. GAPDH serves as the loading control.

(D) Quantification of TRPC3 and TH protein levels, normalized to GAPDH.

Data are presented as mean ± s.e.m., with all data points included. Each data point represents one mouse. Statistical tests: Mann–Whitney test (D, TRPC3). Student's *t* test (B, D-TH). $^{*}P < 0.05$; $^{**}P < 0.01$.

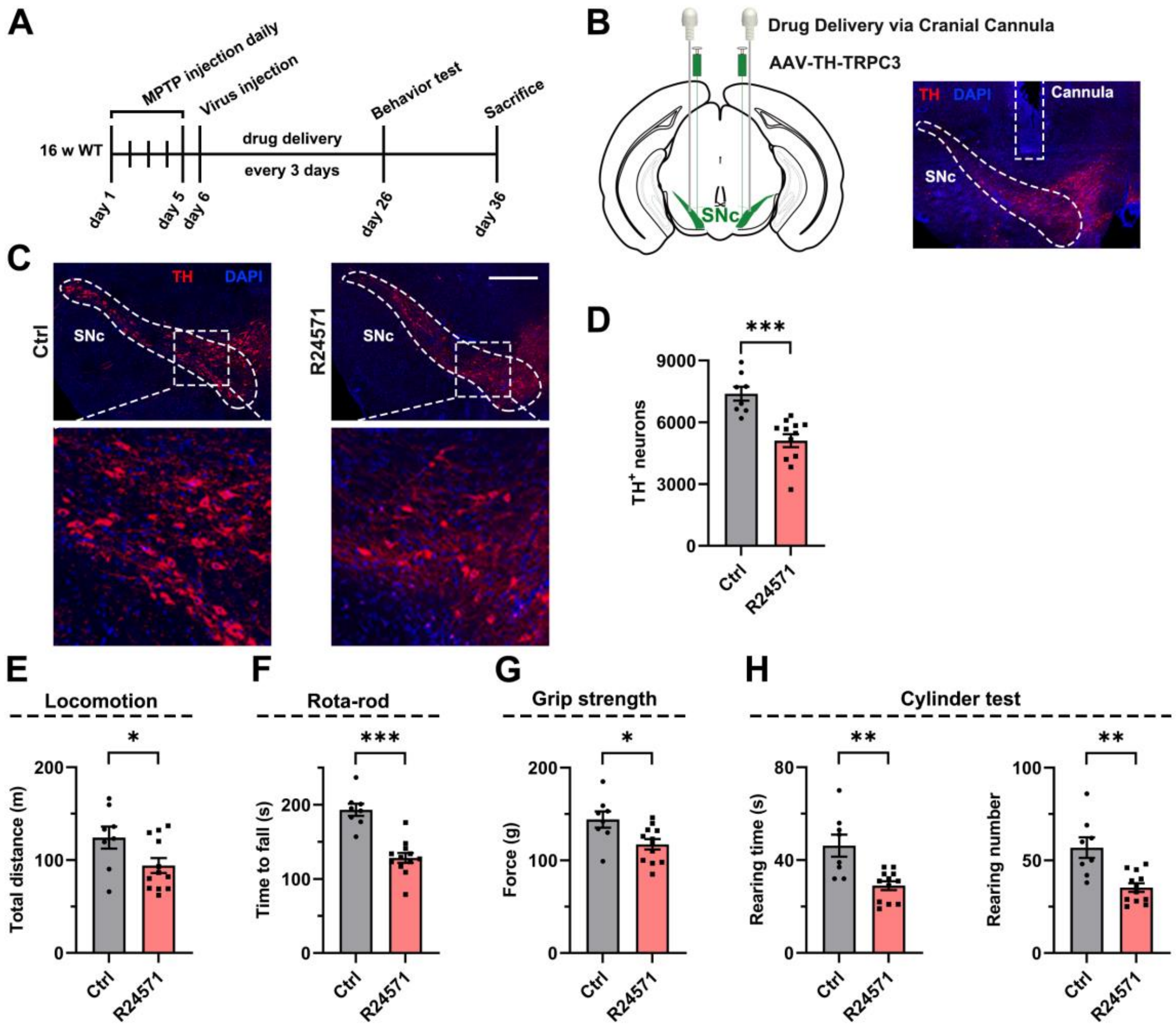

FIGURE S12. Effects of in situ CaM blockade in the SNc on DA neuron survival and behavior in vivo.

(A) Schematic diagram of the experimental timeline.

(B) Left: Schematic of virus injection and cannula placement in the SNc. Right: Immunofluorescence image of a brain slice showing the location of the cannula tip.

(C) Immunofluorescence images of TH^+ neurons in the SNc of TRPC3-overexpressing, MPTP-treated mice, with or without local CaM inhibition (10 µM R24571). Scale bar, 200 µm.

(D) Quantification of surviving TH^+ neurons in the SNc. n = 8, 12 mice per group, respectively.

(E–H) Behavioral assessments: locomotion (E), rota-rod performance (F), grip strength (G), and cylinder test (H).

Data are presented as mean ± s.e.m., with all data points included. Each data point represents one mouse. Student's *t* test. *P < 0.05; **P < 0.01; ***P < 0.001.

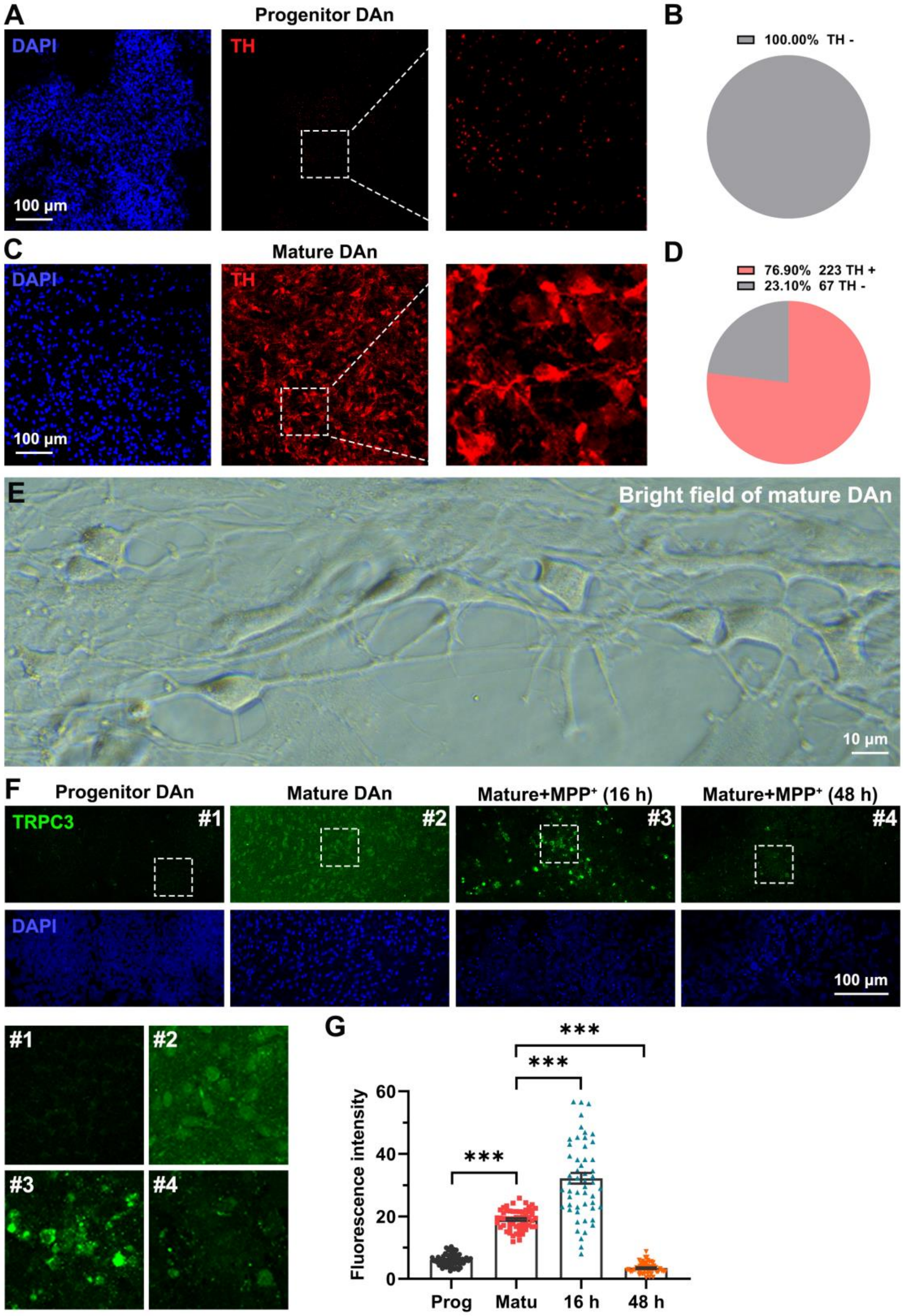

FIGURE S13. Characterization of hESC-derived DA neurons and TRPC3 expression dynamics.

(A, B) Immunofluorescence images (A) and quantification (B) of TH^{+} neurons before

differentiation.

(C, D) Immunofluorescence images (C) and quantification (D) of TH^+ neurons after differentiation.

(E) Bright-field image of differentiated hESC-derived DA neurons.

(F) Immunofluorescence images of TRPC3 expression in undifferentiated cells, differentiated DA neurons, and differentiated DA neurons treated with 45 μM MPP^+ for 16 h or 48 h. Panels (A, C) show cells from a subset of samples in (F) (those under identical conditions), but labeled with different antibodies for distinct proteins.

(G) Quantification of TRPC3 fluorescence intensity under the indicated conditions. n = 61, 58, 54, 56 cells per group, respectively.

Data are presented as mean ± s.e.m., with all data points included. Each data point represents one cell. One-way ANOVA. ***P < 0.001.

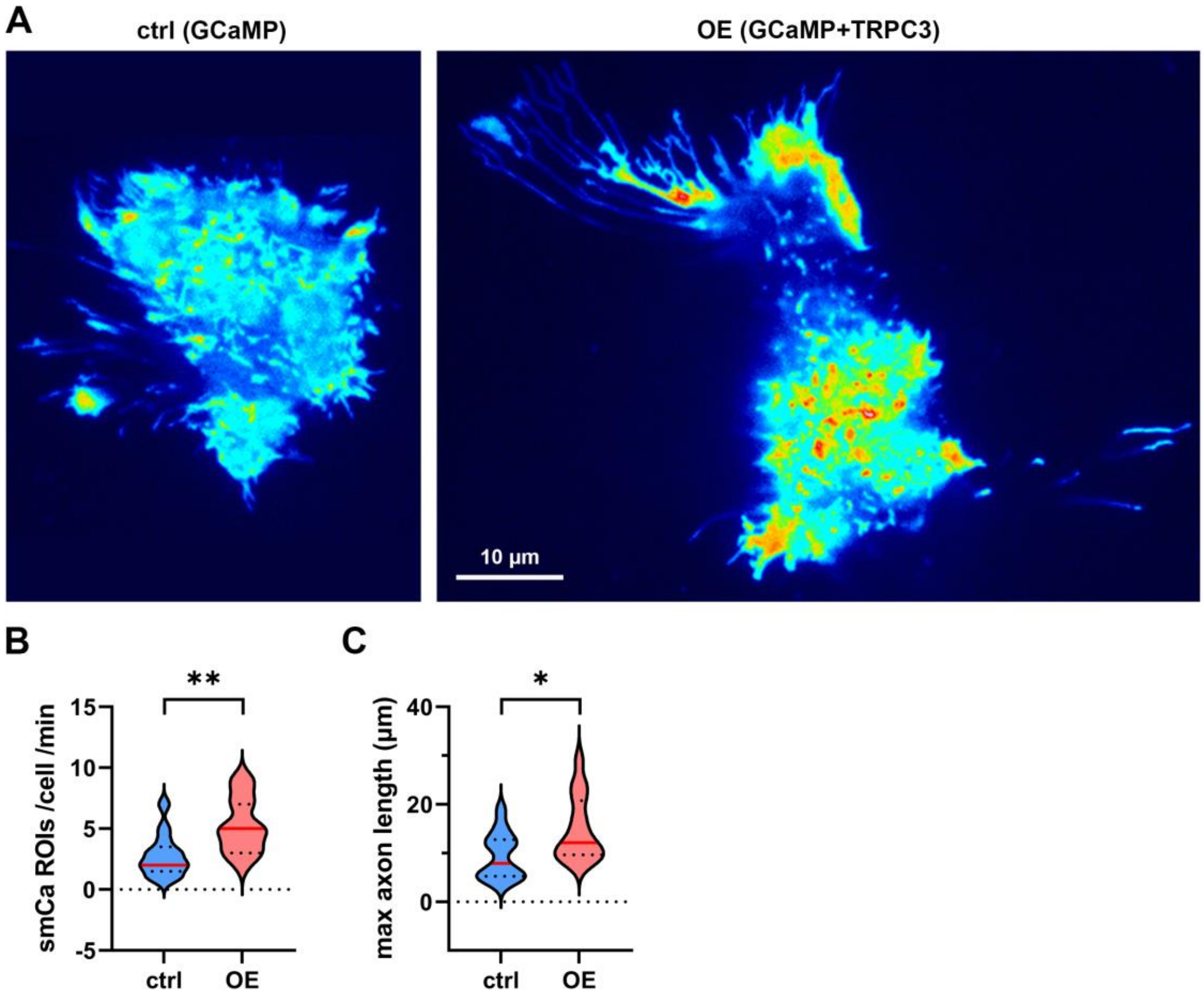

FIGURE S14. TRPC3 overexpression enhances spontaneous microdomain Ca^{2+} activity and neurite outgrowth in SH-SY5Y cells.

(A) Representative TIRF images of SH-SY5Y cells expressing GCaMP alone (control) or together with TRPC3 (TRPC3-OE).

(B) Quantification of spontaneous microdomain Ca^{2+} events, expressed as the number of active ROIs per cell per minute. n = 13 cells for ctrl, 15 cells for OE.

(C) Maximum length of axon-like structures regenerated per cell. n = 13 cells for ctrl, 15 cells for OE.

Data are presented as violin plots with median and quartiles. Statistical tests: Student's *t* test. *$P < 0.05$; **$P < 0.01$.

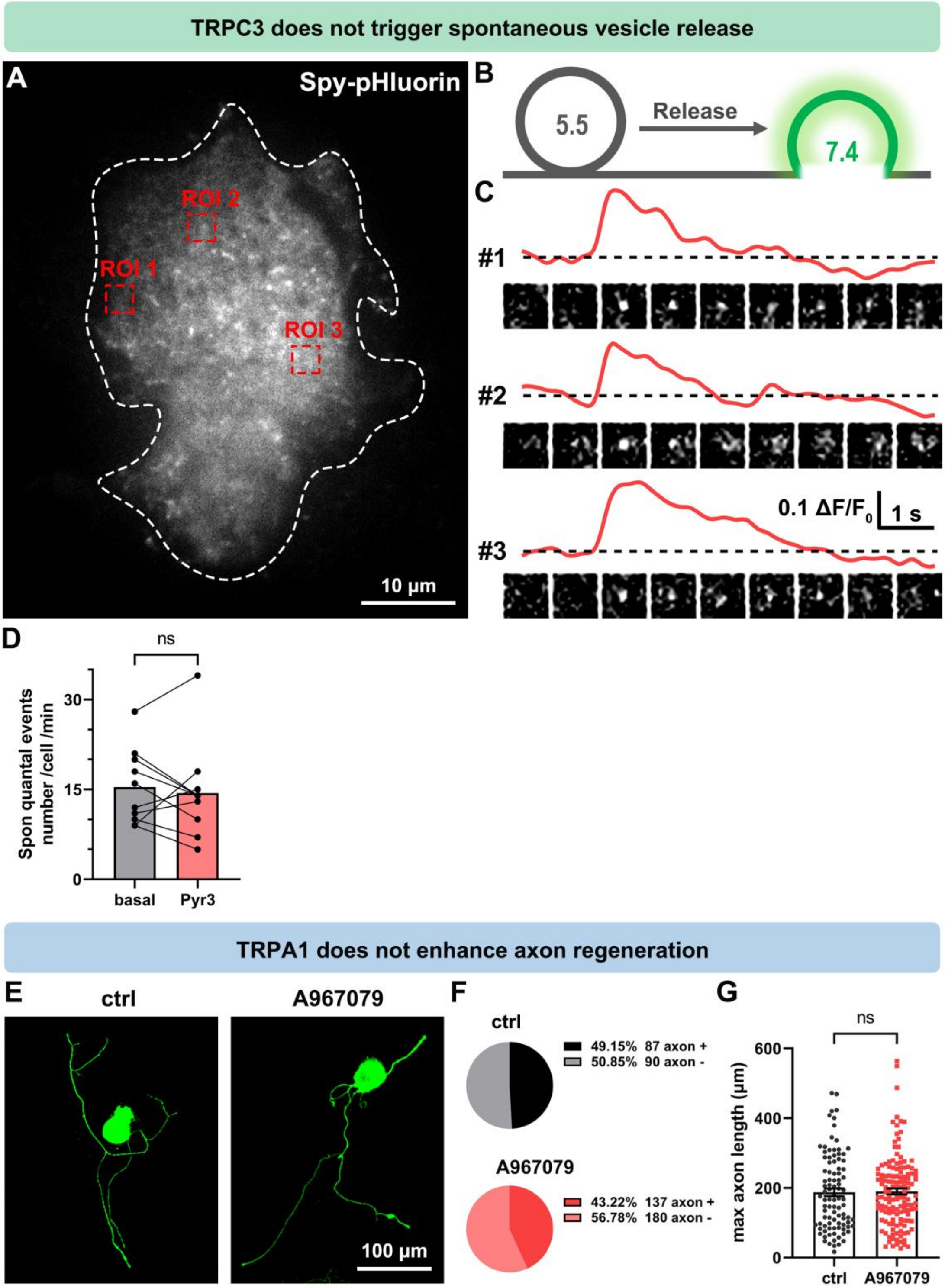

FIGURE S15. TRPC3 does not trigger spontaneous vesicle release, whereas TRPA1 does not promote axon regeneration.

(A) TIRF image of a 24-h cultured DRG neuron transfected with Synaptophysin (Spy)-pHluorin.

(B) Schematic showing that vesicle-membrane-localized Spy-pHluorin emits green

fluorescence when vesicle lumen pH increases from 5.5 to 7.4 during fusion.

(C) Fluorescence intensity over time and TIRF-image montages of three single quantal vesicle release events marked in (A), aligned to the onset of each release. Events occur spontaneously and randomly during a 1-min recording.

(D) Quantification of total spontaneous quantal release events per cell per minute, before and after treatment with 50 μM Pyr3 (selective TRPC3 antagonist). n = 10 cells.

(E) Confocal images of 24-h cultured sensory DRG neurons in control medium or medium containing 1 μM A967079 (selective TRPA1 antagonist).

(F) Percentage of regenerated-axon–positive cells in the control and A967079 groups shown in (E).

(G) Quantification of maximum regenerated-axon length per cell in the groups shown in (E).

Data are presented as mean ± s.e.m., with all data points included. Each data point represents one neuron. Statistical tests: Wilcoxon matched-pairs signed-rank test (D); Mann–Whitney test (G). ns, not significant.

Supplementary Movie Snapshots and Legends:

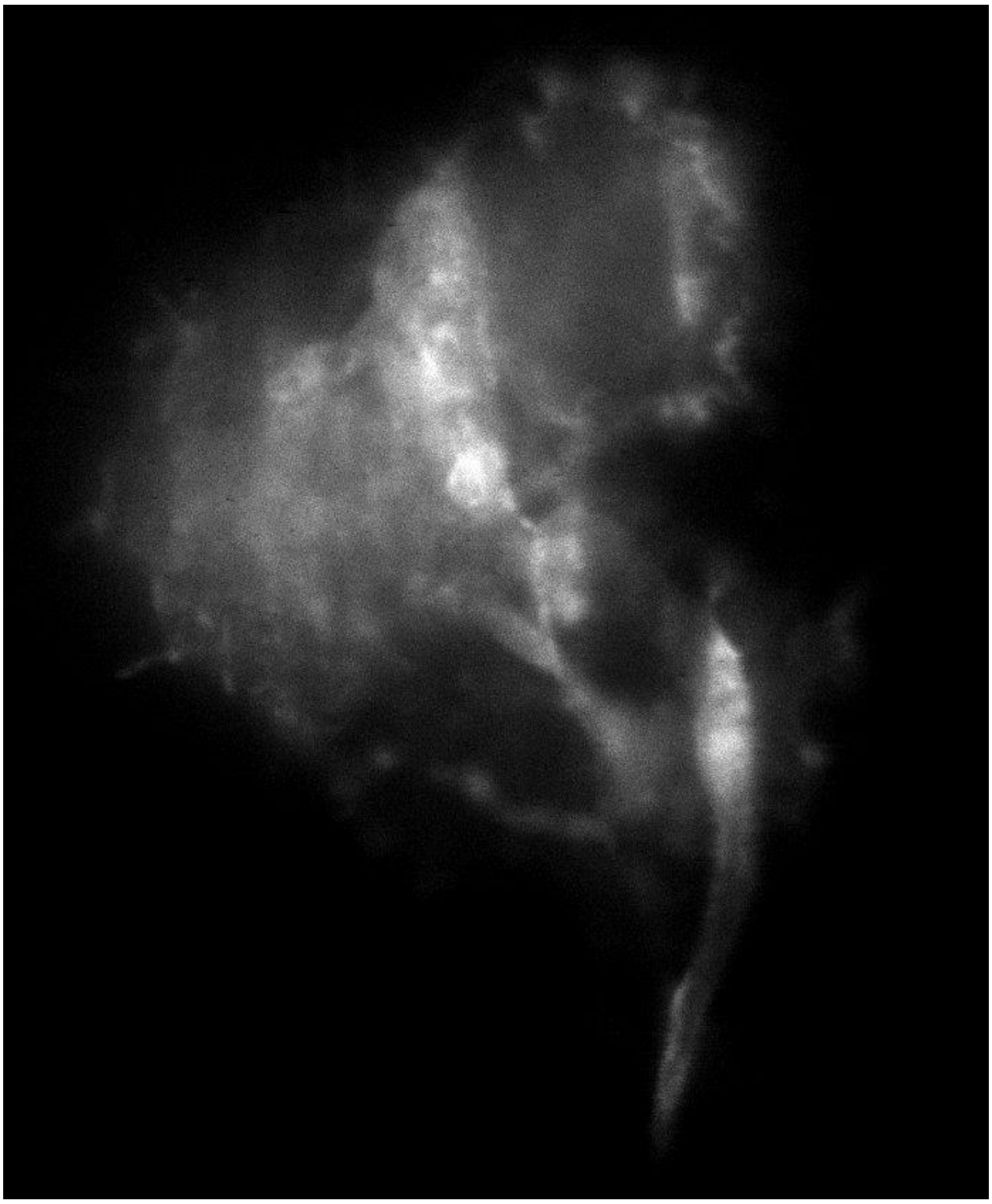

MOVIE S1. Raw TIRF imaging of smCa activity in a DRG neuron.

Raw TIRF recording of a 20-h cultured DRG neuron exhibiting frequent spontaneous microdomain Ca^{2+} (smCa) activity.

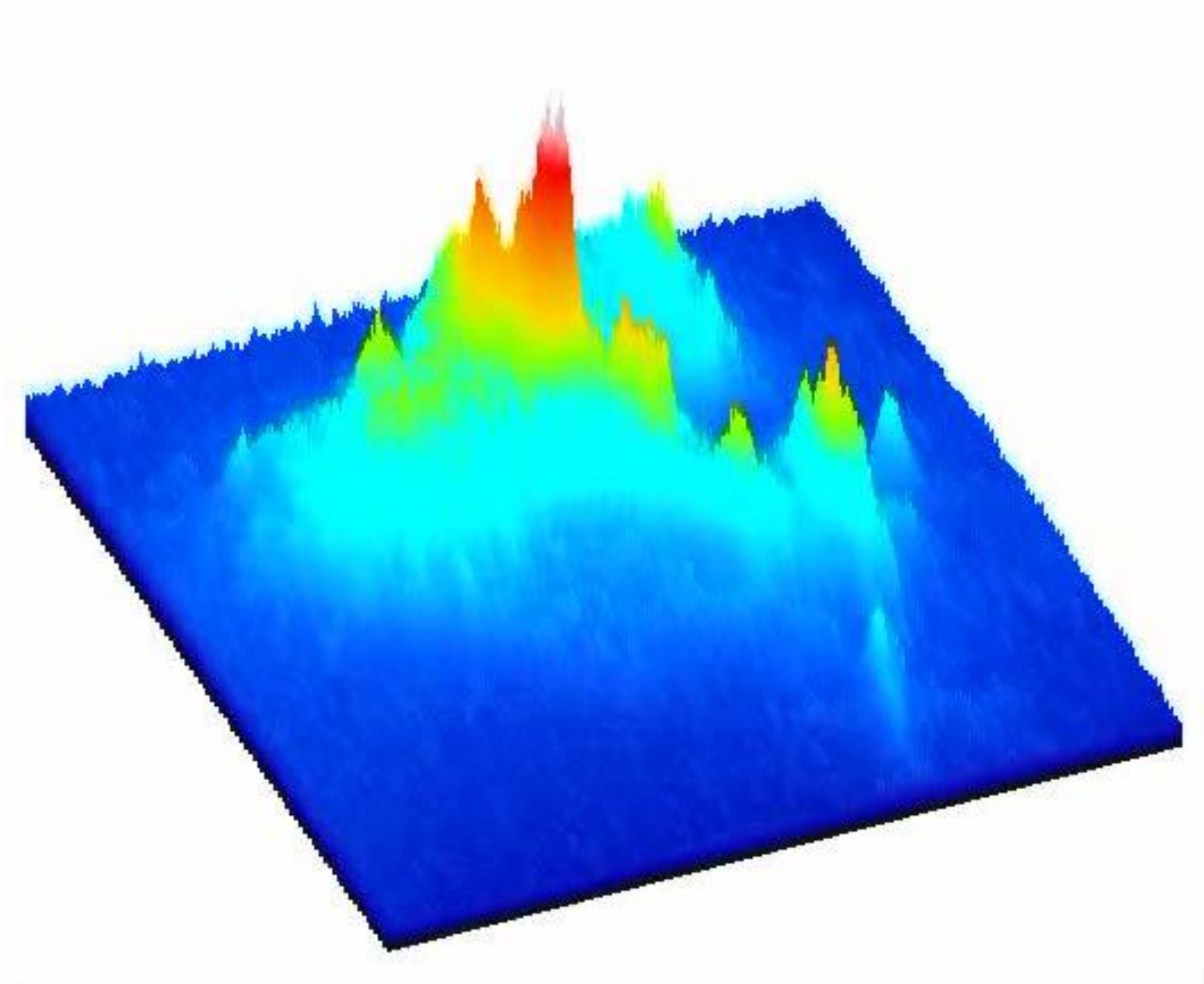

MOVIE S2. Surface plot of TIRF imaging of smCa activity in a DRG neuron.

Surface plot of TIRF recording of a 20-h cultured DRG neuron exhibiting frequent spontaneous microdomain Ca^{2+} (smCa) activity.
