# Supplementary material for: TRPC3‐Driven Calcium Microdomains Instruct Peripheral Nerve Regeneration With Protective Implications for Central Neurons
Source: Adv Sci (Weinh). 2026 Aug 25;13(53):e77156. doi: 10.1002/advs.77156 (PMC13505323; doi:10.1002/advs.77156)
Supplement: Supplementary file 2 [file ADVS-13-e77156-s003.pdf]

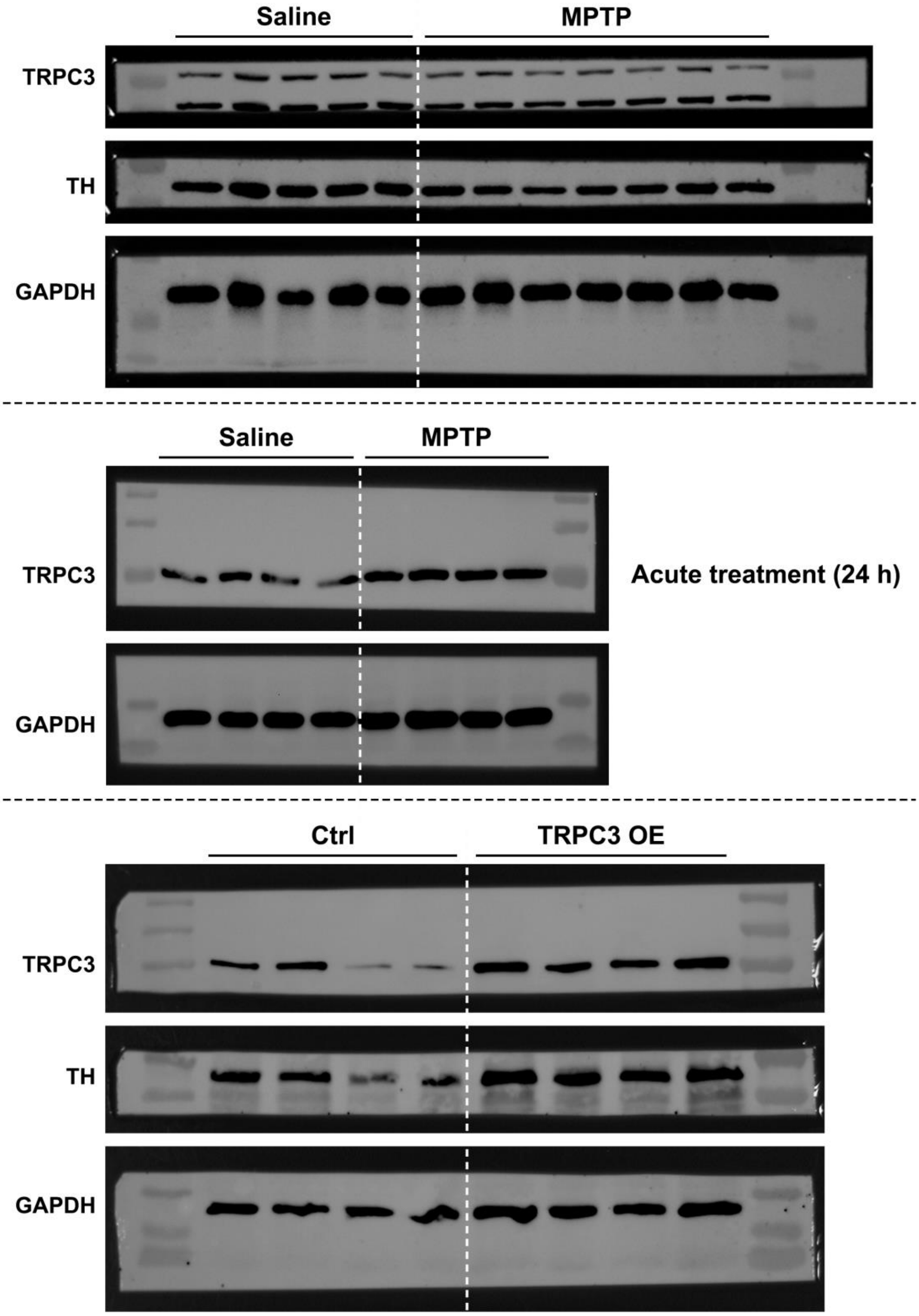
Saline
MPTP
TRPC3
TH
GAPDH
Saline
MPTP
TRPC3
GAPDH
Acute treatment (24 h)
Ctrl
TRPC3 OE
TRPC3
TH
GAPDH

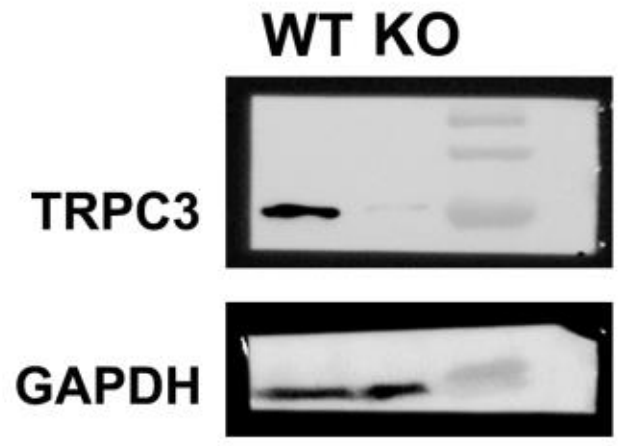
WT KO
TRPC3
GAPDH

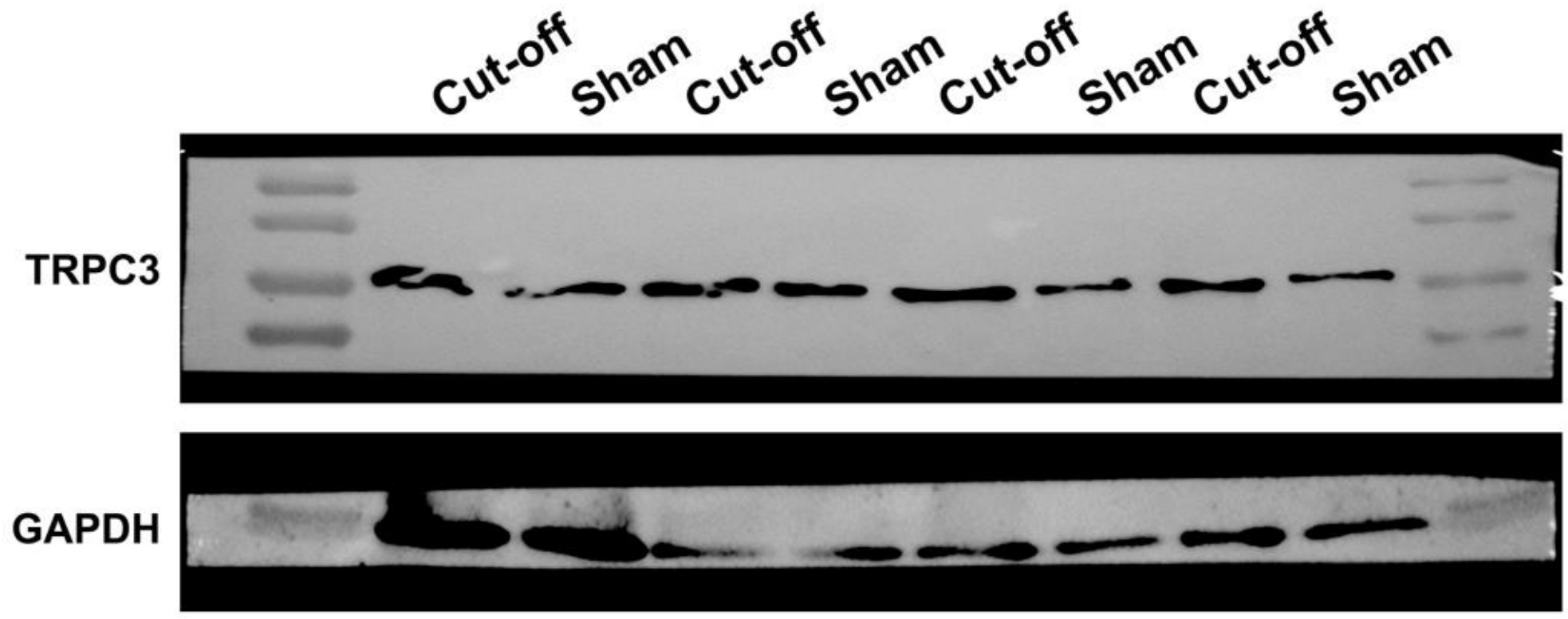
Cut-off
Sham
Cut-off
Sham
Cut-off
Sham
Cut-off
Sham
TRPC3
GAPDH
